# Delayed type I interferon response and the subsequent out-of-sequence cytokine signal inhibit T cell induction in non-surviving Ebola virus-infected patients

**DOI:** 10.3389/fimmu.2026.1806697

**Published:** 2026-04-23

**Authors:** Gang Zhao, Miša Korva, César Muñoz-Fontela, Stephan Günther, Romy Kerber, Sebastian C. Binder, Michael Meyer-Hermann

**Affiliations:** 1 Department of Systems Immunology and Braunschweig Integrated Centre of Systems Biology, Helmholtz Centre for Infection Research, Braunschweig, Germany; 2 Institute of Microbiology and Immunology, Faculty of Medicine, University of Ljubljana, Ljubljana, Slovenia; 3 Bernhard Nocht Institute for Tropical Medicine, Hamburg, Germany; 4 Institute for Biochemistry, Biotechnology and Bioinformatics, Technische Universität Braunschweig, Braunschweig, Germany; 5 Lower Saxony Center for Artificial Intelligence and Causal Methods in Medicine (CAIMed), Hannover, Germany

**Keywords:** cytokine dynamics, innate adaptive immune crosstalk, machine learning - ML, mathematical model, sequential cytokine signaling, Ebola virus, type I interferon

## Abstract

**Introduction:**

While there is evidence that Ebola virus (EBOV) antagonizes the antiviral type I interferon (IFN-I) response, the role of IFN-I for EBOV disease remains controversial, as initially protective responses may contribute to disease pathogenesis later.

**Methods:**

We analyzed patient data from the 2014–2016 West Africa epidemic with a combination of machine learning and mathematical modeling to identify predictive immune mediators and reconstruct their temporal dynamics in survivors and non-survivors.

**Results:**

Our results suggest that IFN-I response in survivors occurs before symptom onset, while non-survivors mount IFN-I responses 3–4 days later, although with a similar strength. This delayed IFN-I response overlaps in time with IL-12 signals in non-survivors. As optimal T cell activation requires a particular temporal sequence in cytokine signals, this impairs the development of T cell-based cellular immunity.

**Discussion:**

The presented patient data analysis helps reconcile the seemingly contradictory role of IFN-I in EBOV disease from a cytokine dynamics perspective and supports the theory of sequential T cell activation, according to which a dysregulated temporal sequence of cytokine signals keeps T cells unresponsive to the pathogen.

## Introduction

Type I interferons (IFN-I) are key antiviral cytokines that induce an antiviral state in infected and bystander cells (1), boost antigen presentation (2), promote NK (3) and T cell (4–6) responses, and enhance antibody responses (7). Ebola virus (EBOV) as well as other filoviruses inhibit various aspects of IFN-I pathways due to the potent IFN antagonist proteins encoded in the virus genome (8–11). Therefore, a high level of IFN-I in EBOV infections might be considered as a potential indicator of an intact immune response. However, the role of IFN-I in the course of Ebola virus disease (EVD), according to currently available data, is controversial (12). Elevated levels of IFN-α were associated with fatal outcomes in humans (13, 14), while high levels of IFN-β were associated with moderate EVD in humans (15). In addition, data from laboratory nonhuman primates (NHP), which are the gold-standard animal models in studying EVD, seem contradictory as well: while high levels of IFN-α/β were associated with fatal outcomes in EBOV infected NHP (16, 17), exogenous IFN-β prolonged survival of EBOV infected NHP (18) and enhanced the efficacy of monoclonal antibodies in rescuing infected NHP (19).

The IFN-I response is highly dynamic (20, 21), and the effect of IFN-I can be diametrically opposite, i.e. immunosuppressive vs. immuno-stimulating, depending on timing (22–24), duration (25), strength (22) or expression of the involved transcription factors (26). Here, we hypothesize that the controversial role of IFN-I in EVD can be explained by the dynamic features of IFN-I responses, and that the early IFN-I response before symptom onset plays an important role in limiting viral replication as well as in shaping the subsequent adaptive immune response. This initial IFN-I response remains difficult to measure in practice.

We analyzed patient data from the 2014–2016 West Africa EBOV epidemic (14) by a combination of machine learning and mathematical modeling approaches. In a first step, highly predictive factors were identified by a machine learning approach. IFN-β was found as one of the factors associated with fatal outcomes, confirming previous studies based on the same data set (14) with a different approach. The common subunit of the IL-12 family of cytokines, i.e. IL-12p40 (referred to IL-12 hereafter), and RANTES, which is a potent chemoattractant factor for T cells, are among the factors that are associated with survival. Both IL-12 and RANTES are essential for the activation of CD8+ T cells in anti-viral response (27, 28). In the second step, the dynamics of a subset of the predictive factors were investigated in a mechanistic model which integrates current knowledge of the relevant immune regulations. The modeling analysis found that EVD survivors had an early but transient IFN-β peak about 5 days before symptom onset while non-survivors had this peak only 1–2 days before symptom onset.

The modeling analysis revealed a clear temporal sequence of cytokine induction in survivors, i.e. IFN-β followed by IL-12. This sequence was lost in non-survivors. Systemic IFN-I induces the migration and maturation of antigen presenting cells (2, 29). At the same time, both IFN-I and IL-12 are essential cytokines for T cell priming in a variety of contexts (30), which define a future polarization of naive T cells into a Th1 phenotype. In light of the importance of the right sequence of activation signals in T cell induction (24, 31, 32) and the importance of IFN-β and IL-12 in inducing anti-viral cellular immunity (29, 33), we hypothesized that T cell activation in EVD non-survivors is characterized by a disrupted sequence of signals, which contributed to their exhausted phenotype (34). We tested this hypothesis by a second modeling approach, in which three parallel models of T cell activation by cytokines were evaluated in terms of their ability to predict IFN-γ data in the same patient cohort. The sequential model was successful while the non-sequential and the reverse-sequential model failed. To the best of our knowledge, this data-driven modeling work provides the first mechanistic demonstration, using patient data, of the ambiguous role of IFN-I in EVD (35) from the perspective of cytokine dynamics and supports the theory of sequential T cell activation (31, 32).

## Methods

The data set as well as details on sample collection and bioanalytical methods were previously reported (14). This study included 249 samples measured on the same day while samples measured on other days (n=137) were excluded in order to avoid batch effects. The demographics, initial viral load, sample characteristics, as well as participation in the favipiravir trial are summarized in **Supplementary Table 1**. A sanity check procedure removed 71 samples due to lack of key information or marginal sampling time (defined as days post symptom onset, DPO) and 14 predictors due to a high portion of missing or identical values. The outlier detection procedure removed 6 samples.

The present analyses were composed of two steps. At first, the importance of features (predictors) in predicting final EVD outcome was evaluated by a multivariate analysis. An ensemble of elastic-net penalized logistic regressions was employed. The ensemble consisted of 1000 randomly chosen training (80%) and testing (20%) subsets of the whole data set. The elastic-net parameter α was set to 0.95, which effectively reduced the number of predictors when there is (multi-)collinearity while avoiding numerical instability associated with large values of α (for example 0.99 or 1). The training was done with 4-fold cross-validation. Regression accuracy was assessed by the area under the receiver operating characteristic curve (ROC-AUC) (**Supplementary Figure 1A**). To generate a robust estimation of feature importance over the whole data set, regressions for which the ROC-AUC was out of the inter-quartile range were excluded from further analysis (**Supplementary Figure 1B**). The frequency of appearance for a particular predictor was regarded as indicator of its importance. A threshold for the frequency of appearance of a predictor of 50% was applied to define important predictors. The above analyses were done on immune and coagulation/endothelial factors, respectively, with Ct (cycle threshold) being a global predictor, i.e., it was included in the analysis of both immune and coagulation/endothelial factors.

Ordinary differential equation (ODE) models were built and fitted to the temporal profiles of relevant cytokines/chemokines. The days post infection (DPI) were reconstructed from DPO by assuming an average incubation period of eight days (36). The temporal profile of the cytokines/chemokines were generated by a weighted bootstrap scheme where samples of the neighboring days were included with a reduced weight, in generating the bootstrap mean and standard deviation. The fitting procedure was guided by minimizing the root mean square error (RMSE):


$$RMSE=\sqrt{\sum_{i=1}^{N_{s}}\;\frac{{\left(y_{s,i}-{\hat{y}}_{s,i}\right)}^{2}}{\sigma_{s,i}^{2}}+\sum_{j=1}^{N_{n}}\;\frac{{\left(y_{n,j}-{\hat{y}}_{n,j}\right)}^{2}}{\sigma_{n,j}^{2}}}$$

where, suffix s and n denote survivors and non-survivors, respectively, N denotes the number of data points, y and ŷ denote simulated result and the bootstrap mean, respectively, and σ the standard deviation associated with the bootstrap mean. The identifiability property of the fitted parameters was explored by Markov Chain Monte Carlo (MCMC) sampling, which was set to start near the best fit result.

The ODE models [text files used in SBPD Toolbox (37)] and the R scripts for data sanity check, outlier detection, ensemble regression [using mlr3 ecosystem (38) and glmnet package (39, 40)], and weighted bootstrapping are included in the supplementary files.

## Results

### Fate predictive factors

**Figure 1:**
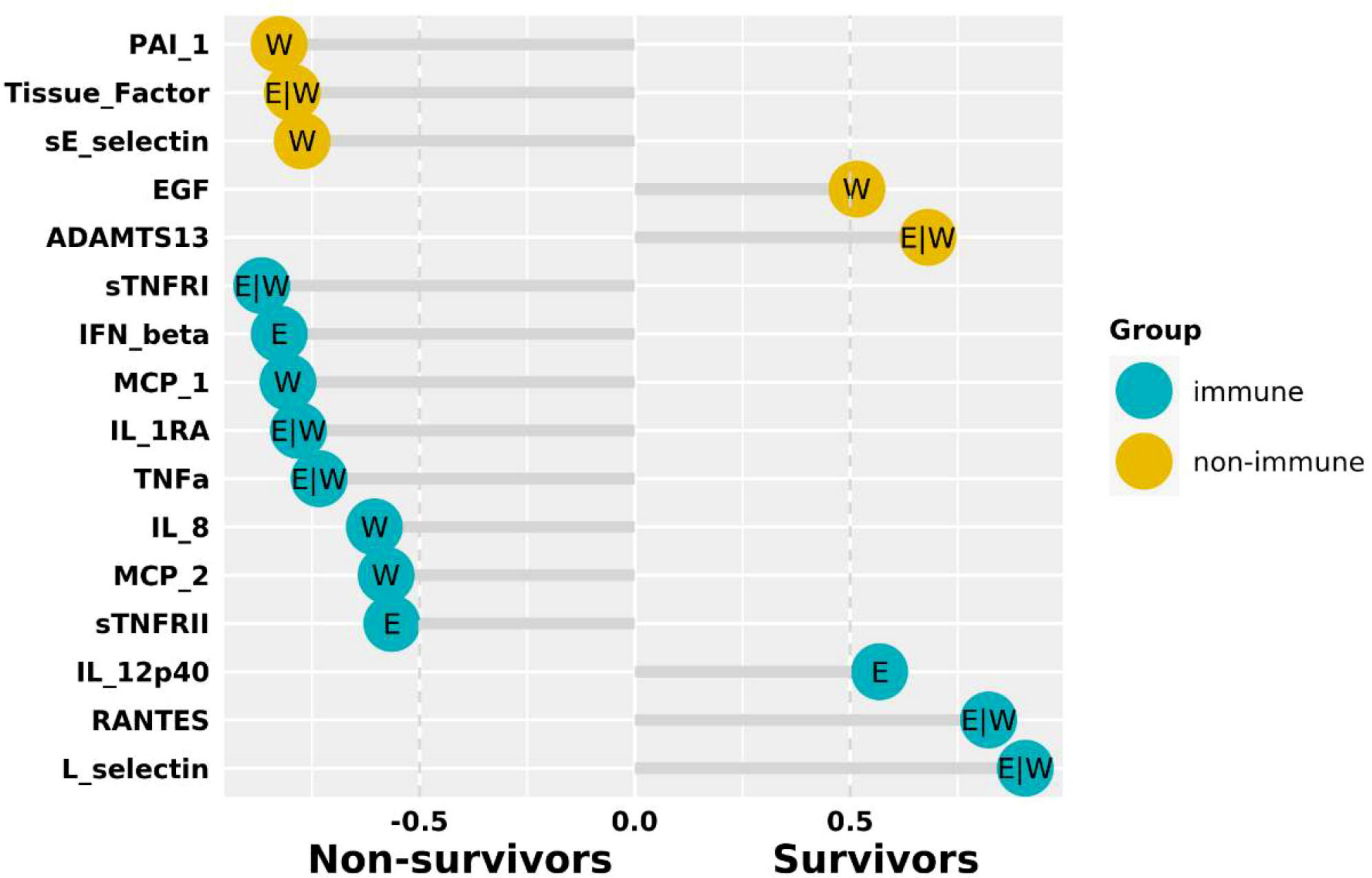
The importance of fate predictive factors obtained from the ensemble regression on samples in the early time window only (E: DPO<=5), as well as the whole data set (W: DPO<=11). Factors associated with survivors are shown in the positive region while those associated with non-survivors in the negative region. A threshold of 0.5 in the frequency of appearance was applied (see Methods). The analyses were performed on immune and non-immune mediators separately, with Ct (not shown) being the most important factor in both cases.

The ability of each soluble mediator in discriminating survivors from non-survivors has been evaluated previously (14). Here a multivariate analysis was performed on both early (DPO ≤ 5) and whole (DPO ≤ 11) samples (see Methods). Among the identified important factors IFN-β, IL-12 and sTNFRII were important only in the early time window (**Figure 1**), suggesting complex dynamics.

Next, we generated temporal profiles (see Methods) for the identified important immune mediators and found two distinct patterns. For IFN-β and IL-12, the curves of the two patient groups cross each other, i.e., the relationship between survivors and non-survivors switch over time (**Supplementary Figure 2**). RANTES (CCL5) showed only a trend to such a switch (**Supplementary Figure 2**). This indicated complex mechanisms underlying the induction of these cytokines/chemokines. Other important immune mediators had clear boundaries between survivors and non-survivors (**Supplementary Figure 3**), suggesting that the difference of these immune mediators between survivors and non-survivors were largely explained by the difference in viral load.

### A dynamic model of cytokine responses in EVD

**Figure 2:**
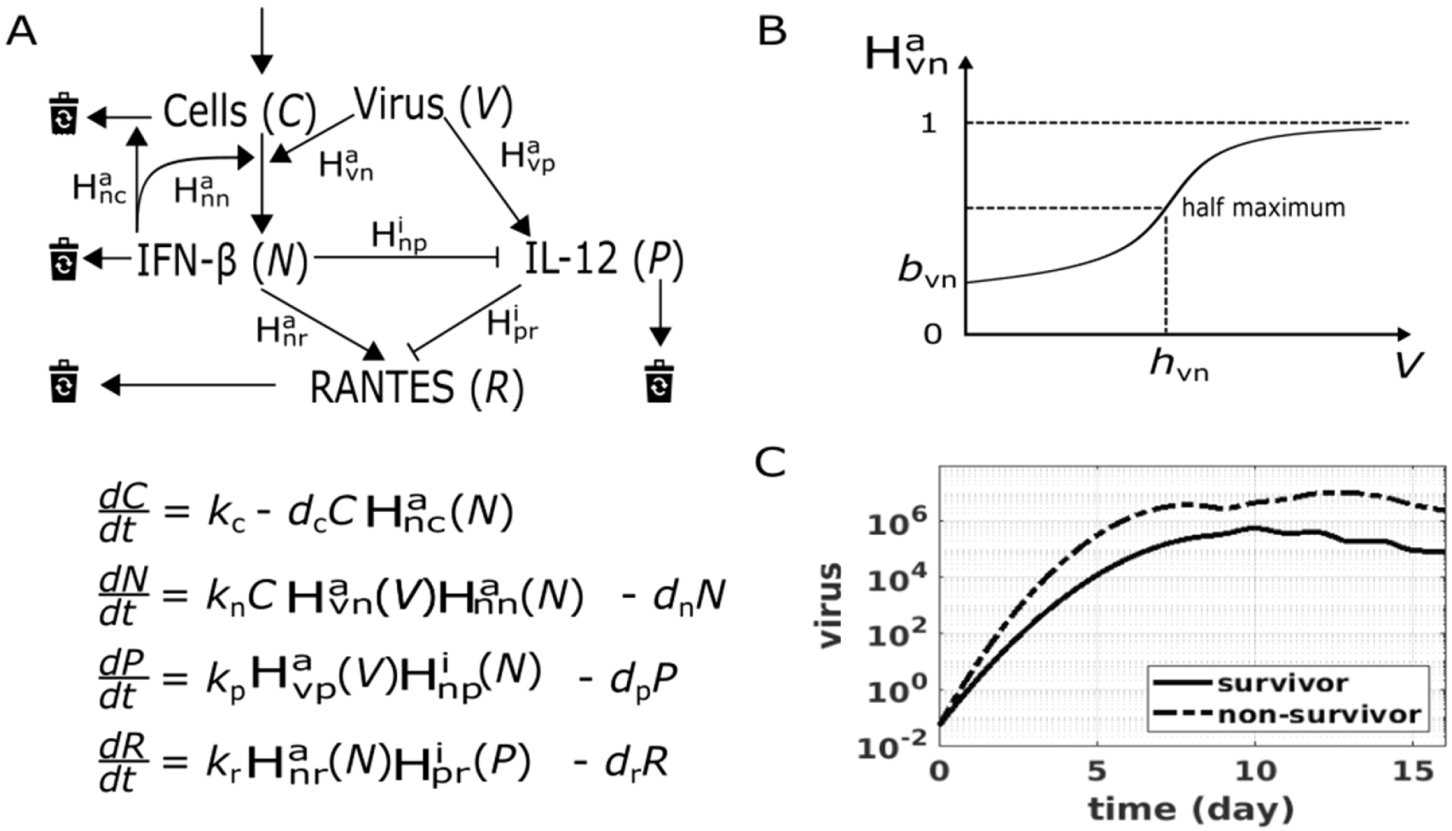
The mathematical model of cytokine/chemokine responses in EVD. **(A)** Model scheme and equations. Arrows denote activation, bar ends denote inhibition. Virus induces IFN-β secretion from infected and bystander cells (*C*). IFN-β augments its own secretion from *C* and induces the apoptosis of *C*. Virus induces, while IFN-β inhibits, IL-12. RANTES is induced and inhibited by IFN-β and IL-12, respectively. The Hill functions in the equations accompany the corresponding arrows in the scheme. The superscripts of Hill functions denote activation (a) or inhibition (i). The first and the second subscript of Hill functions denote the source and the target of the interaction, respectively. **(B)** An example Hill function, representing the effect of virus (*V*) on IFN-β (*N*) induction, together with its two parameters (*b*_vn_ and *h*_vn_). **(C)** Interpolated viral dynamics, employed as input to the model. Day zero denotes the time of infection. Parameter values are provided in **Supplementary Table 2**.

We developed a mechanistic mathematical model for the dynamics of IFN-β, IL-12 and RANTES in EVD patients (**Figure 2A**). The cellular source of IFN-β was explicitly modelled (Cells in **Figure 2A**) whereas the cellular producers of other cytokines are not individually represented. This choice reflected the central regulatory role of IFN-I and the strong experimental evidence for its dynamic interplay with its cellular source, which includes infected and bystander cells, especially various myeloid cells, particularly dendritic cells (DC). In the model, a generic DC population is assumed to account most of the IFN-I production. IFN-I feedback signaling through the IFN receptor is required for DC maturation and migration, activation, and IFN-I production *in vivo (*2, 41–43). These mechanisms were modelled by a Hill function H^a^nn (**Figure 2A**). On the other hand, function and number of DCs are regulated during acute viral infections, mainly due to activation-induced exhaustion and the activation of the intrinsic apoptosis pathway by IFN-I (36, 44). These interactions were incorporated via a second Hill function H^a^nc (**Figure 2A**). An exemplary Hill function and its parameters are shown in **Figure 2B**. The production of IL-12 and RANTES was represented phenomenologically through Hill functions that summarize upstream regulatory influences.

The temporal profiles of viral load were generated by linear interpolation along the weighted bootstrap mean Ct values, for survivors and non-survivors, respectively (**Figure 2C** and Methods). The interpolated viral dynamics entered the model as inputs and drove the immune response via Hill functions H^a^vn and H^a^vp (**Figure 2A**). The dynamics of IL-12 and RANTES were modelled in a phenomenological manner: their cellular sources were not explicitly modelled while their regulations on various levels were lumped into Hill functions. These included the inhibition of IL-12 by IFN-β (26, 29, 45–50) (H^i^np in **Figure 2A**), the inhibition of RANTES by IL-12 (51) (H^i^pr in **Figure 2A**) and the induction of RANTES by IFN-β (52) (H^a^nr in **Figure 2A**).

### Differential IFN-I responses shape the immune responses in survivors and non-survivors

**Figure 3:**
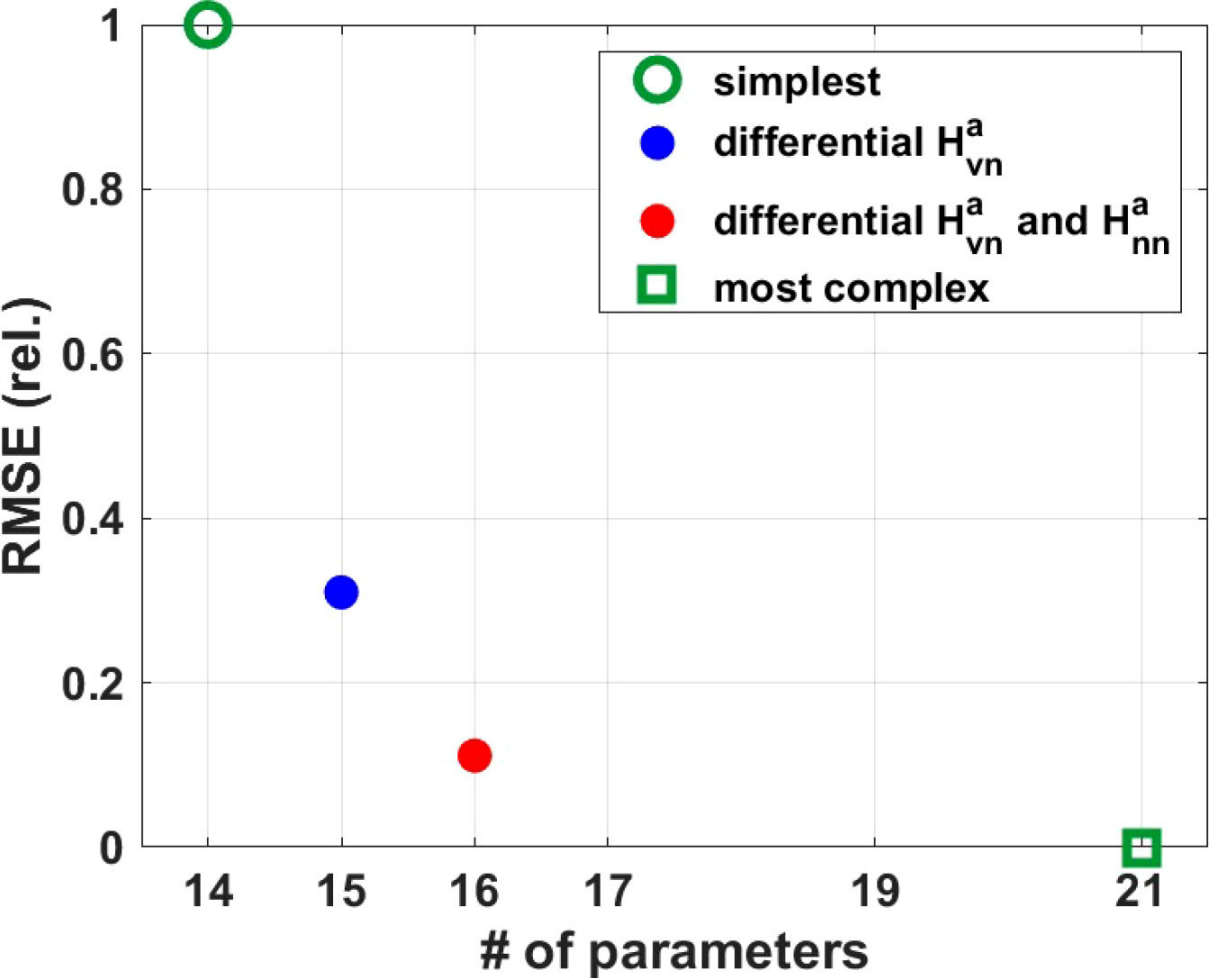
A minimal set of differential immune pathways that can explain the immune response in EVD survivors and non-survivors. The RMSE (vertical axis) is linearly rescaled so that the RMSE of the most complex model is zero and that of the simplest model is one, i.e. RMSE_rel= (RMSE - RMSE_min)/(RMSE_max - RMSE_min).

We aimed at targeting a minimal set of immune pathways (**Figure 2**) that can explain the differential cytokine response between survivors and non-survivors. We started by investigating two extreme scenarios. In the simplest scenario, survivors and non-survivors were simulated with the same parameters where only their viral dynamics (implemented as inputs) were different. In the most complex scenario, all the seven immune pathways, i.e. the position of the half maximum value (see **Figures 2B, C**) of the seven Hill functions, were allowed to be different between survivors and non-survivors during parameter optimization. The relative RMSE (see Methods) from the simplest and the most complex scenario are shown in **Figure 3** (green circle and square, respectively) and defined a space in which we further explored the contribution of each immune pathways by its impact on the RMSE value. We found that differential IFN-β induction by virus (H^a^vn) accounted for 70% of the relative RMSE (blue **Figure 3**). Among the other six immune pathways, differential IFN-β positive feedback on its cellular source (*C*), i.e. H^a^nn, reduced the relative RSME value most (~20%, red **Figure 3**). This suggested that differences in IFN-β induction from its cellular sources largely shaped the observed differences of the immune response in EVD survivors versus non-survivors. The model with differential H^a^vn and H^a^nn is denoted “minimal model” in the following and was investigated further.

### Dynamic difference in immune responses between survivors and non-survivors

**Figure 4:**
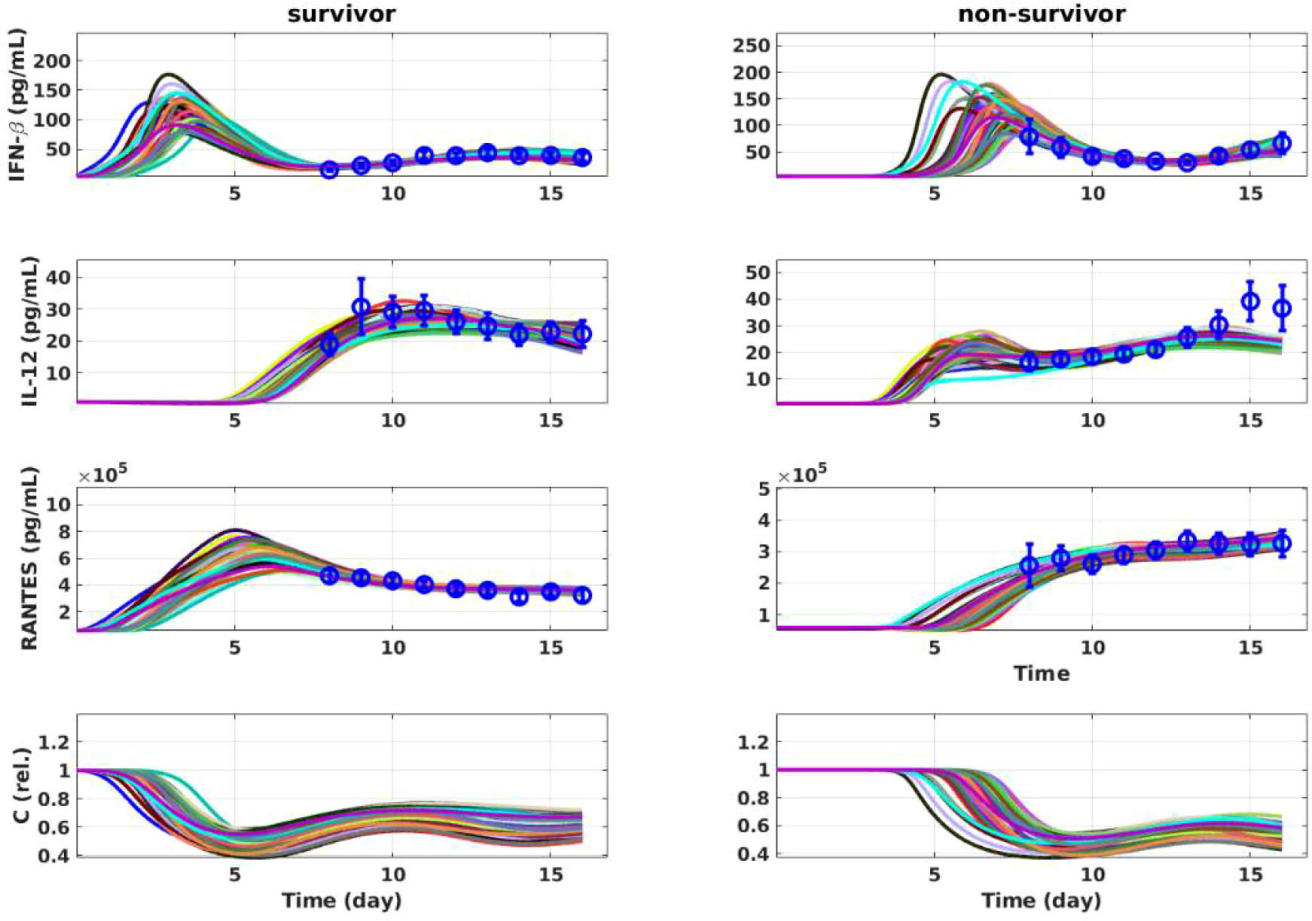
Simulation of the minimal model. The best fit result, together with ninety-nine randomly selected simulations from the MCMC sampling, are shown with different colors. Data are presented by circles with error bar. Day 0 denotes the time of infection.

The minimal model predicted two immune signatures that distinguished survivors from non-survivors before symptom-onset, although a limited level of variation existed within each group (**Figure 4**). Firstly, IFN-β peaked about 4 days earlier in survivors than in non-survivors, while the strength of the response was similar in the two groups. Secondly, there was a well-defined temporal sequence between IFN-β and IL-12 induction in survivors, while in non-survivors, this temporal sequence was lost such that the two cytokines were largely concurrent.

### Patient cytokine dynamics support a sequential model of T-cell activation

Antigen-dependent T cell activation requires T cell receptor activation by antigen (signal 1), co-stimulatory signals (signal 2) and cytokine induced signals (signal 3). The right sequence of three activation signals is vital for the induction of T cell based cellular immunity (24, 31). The distinct temporal sequence between IFN-β and IL-12 in EVD survivors and non-survivors, as predicted by the minimal model, suggested that the development of cellular immunity was impaired in EVD non-survivors due to the time-overlap of IFN-β, which is expected to promote T cell receptor signaling and costimulation, and IL-12 as an example of signal 3 cytokines required for CD8+ T cell activation.

**Figure 5:**
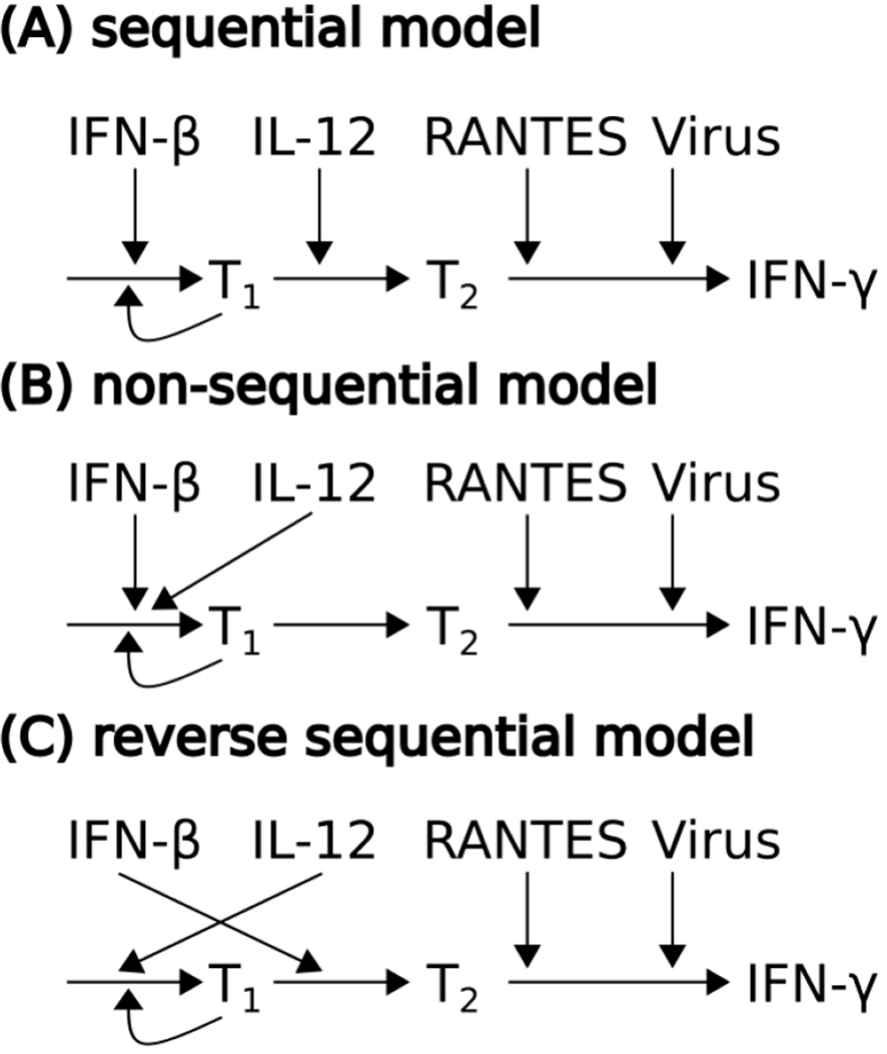
Models of T cell activation and IFN-γ secretion. Activated T cells (T_1_) either undergo proliferation or differentiate into effector cells (T_2_), which are then guided by the chemokine RANTES to the infection site and secret IFN-γ when they encounter virus infected cells. Current knowledge of T cell activation supports the sequential model **(A)** where IFN-β induces the activation and proliferation while IL-12 induces the differentiation into effector cells. Two other models **(B, C)** were generated by adjusting the sequence of IFN-β and IL-12, in order to test the uniqueness of the (canonical) sequential model.

**Figure 6:**
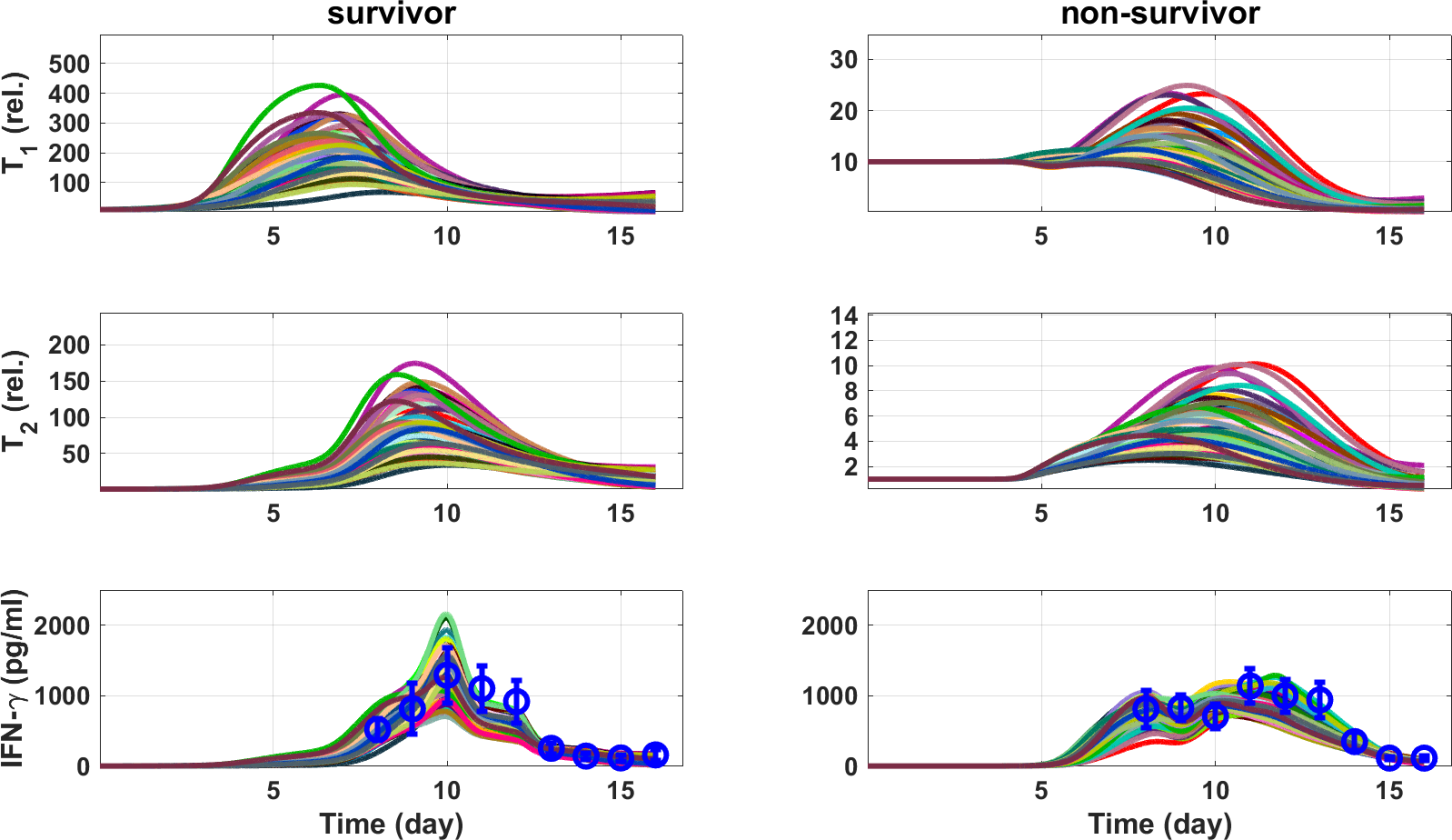
Simulation of the sequential model for T cell activation and IFN-γ secretion. The best fit result, together with ninety-nine randomly selected simulations from the MCMC results, are shown with different colors. Data are presented as circles with standard deviation. T₁ and T₂ are activated T cells and effector T cells, respectively.

We therefore tested whether the theory of sequential signals in T cell activation was consistent with the data set of EVD patients. In particular, the temporal profile of IFN-γ of both survivor and non-survivors were fitted by three mathematical models, each representing a distinct sequence of cytokine signals in T cell activation (**Figure 5**). In a sequential model (**Figure 5A** and **Supplementary Equations S1-S3**), naive T cells are activated and undergo proliferation (T_1_) in response to IFN-β exposure. IL-12 triggers the transformation from proliferating cells to an effector phenotype (T_2_). The effector cells are guided by the chemokine RANTES to the sites of infection, where they secrete IFN-γ upon encounter of infected cells. The time course of IFN- β, IL-12, RANTES from the minimal model, as well as the temporal profile of virus, were employed as inputs. By adjusting the sequence of the two cytokines in the sequential model, two other models of T cell activation were generated (**Figures 5B, C**). In the non-sequential model (**Figure 5B** and **Supplementary Equations S4-S6**), the two cytokines both activate the proliferation process, while in the reverse sequential model (**Figure 5C** and **Supplementary Equations S7-S9**), the role of the two cytokines were reversed. The sequential model was successful in explaining, simultaneously, the IFN-γ response in survivors and non-survivors (**Figure 6**), while the other two models failed (**Supplementary Table 3**).

The simulation of the sequential model suggested that both proliferating (T_1_) and effector (T_2_) T cells in survivors were more than 10-fold higher than in non-survivors (**Figures 6A, B** vs **D, E**). However, the levels of IFN-γ were comparable in both groups (**Figures 6C** vs **F**). Considering a 10 to 100 fold higher viral load in non-survivors (**Figure 2C**) and 10 fold lower activated T cell numbers (**Figure 6**) a single activated T cell in non-survivors was exposed to 100 to 1000 fold more antigen and secretes more than 10 fold IFN-γ, as compared to a single T cell in survivors, indicating that activated T cells in non-survivors are more likely to undergo activation induced cell death. This per-cell T cell hyperactivation can be quantified in the model by the ratio of IFN-γ output to effector T cell number (IFN- γ/T_2_), which is substantially elevated in non-survivors relative to survivors. This “workload” metric captures the qualitative state of a small pool of T cells operating under extreme antigen burden — consistent with the T cell exhaustion phenotype directly observed in fatal EBOV infection (34). The model thus captures the functional cause (per-cell hyperactivation) that aligns with the clinically observed result (exhaustion), providing independent support for its biological plausibility.

## Discussions

We analyzed patient data from the 2014–2016 EBOV outbreak in West Africa, by classical machine learning (ML) approaches, and identified soluble mediators that are highly predictive of the patients’ fate. Strikingly, IFN-β and IL-12, two critical cytokines in the defense against viruses (29), were associated with the survival of patients. The longitudinal profile of the two cytokines in non-survivors and survivors exhibited a switch (**Supplementary Figure 2**). The opposition between the two cytokines was explained when the relevant data was analyzed with dynamic knowledge-based mathematical models that took the timing of the relevant immune processes into account. While the ML approach was successful in identifying the biomarkers for predicting disease outcome, the knowledge-based dynamic modelling approach linked those biomarkers to the current understanding of the relevant immune responses. The work presented here demonstrated that the combination of the two methods provided a deeper understanding of EVD disease progression. We emphasize that the mathematical modeling approach serves a hypothesis-generating role: it demonstrates that the observed patient cytokine dynamics are consistent with established immunological mechanisms, and it generates experimentally testable predictions – for example, that deliberate advancement of the timing of IFN-I exposure in animal models should improve T cell responses and survival outcomes.

A key step in the mathematical modeling approach was to include the cellular source of IFN-I. In the model, the variable C represents a composite innate IFN-producing cell compartment, with plasmacytoid dendritic cells (pDCs) likely being the primary cellular archetype given their documented role as the most potent type I IFN producers in response to viral pathogen associated molecular patterns (PAMPs). However, the model structure is equally applicable to other IFN-producing innate cell populations — including inflammatory monocytes, macrophages, and classical dendritic cells — all of which contribute to IFN-β production during EBOV infection. The kinetic interactions modelled for C, i.e. a motif with IFN-β positive feedback (H^a^_nn_) and activation-induced depletion (H^a^_nc_), are consistent with mechanisms documented across this broader innate cell compartment. The implemented interplay between IFN-I and its cellular source (**Figure 2A**) allowed the model to generate transient wave(s) of IFN-I, largely before (self-reported) symptom onset (**Figure 4**). While no data was observed during this period in humans, data from mouse and non-human primate models showed early and robust IFN responses before the appearance of circulating virus (53, 54). In contrast, while viral load activates both IFN-β (H^a^vn) and IL-12 (H^a^vp) through structurally identical Hill terms, IL-12 lacks an equivalent motif, consistent with the absence of established biological evidence for such a mechanism: IL-12 signaling acts primarily on T cells and NK cells. IL-12 dynamics are therefore structurally constrained to follow viral load kinetics and cannot produce early pre-symptomatic transients regardless of parameterization. Importantly, this conclusion is also mathematically necessitated by the observable data at clinical presentation: in survivors, IFN-β levels are already declining at symptom onset while viral load (V) remains high or rising. Reproducing a cytokine trajectory that falls while its primary stimulus rises requires the target-cell depletion mechanism uniquely encoded in the IFN-β and its cellular sources axis. The optimizer’s selection of H^a^vn reflects this biological asymmetry: it is the only pathway in the model with the regulatory architecture capable of explaining the pre-symptomatic IFN-β timing difference observed between survivors and non-survivors. In addition, the MCMC credible intervals for H^a^vn (**Supplementary Table 2**) show that survivor and non-survivor values separated by nearly four orders of magnitude, confirming that the dominance of H^a^vn is not sensitive to parameter perturbations.

Systemic IFN-I from infected and bystander cells leads to the migration and maturation of dendritic cells that can present antigen (signal 1) and provide costimulatory signals (signal 2) for T cell priming (2, 29, 43, 55–57). Both, IFN-I and IL-12, are signal 3 cytokines for T cell priming in a variety of contexts (30), which define a future polarization of naive T cells into a Th1 phenotype. However, the sensitivity of CD8 T cells to signal 3 appears to depend on the pathogen-induced overall inflammatory milieu (58). It was shown that pre-exposure to IFN-I makes CD8 T cells unable to receive IFN-I as signal 3 (32), suggesting an important role of IL-12 as signal 3 in this context. In addition, there is evidence that CD8 T cells undergo extensive proliferation after antigen (signal 1) and costimulation (signal 2), but only develop the cytolytic effector function after IL-12 exposure, indicating that proliferation and development of effector function are uncoupled (59). Similarly, CD4 T cell differentiation is dependent on the local cocktail cytokine signals produced by innate immune cells (60). In the context of viral infection where Th1 induction is essential, CD4 T cells follow a two-step activation pattern. TCR signals stimulate the Th1 transcription factor T-bet but suppress the expression of IL-12 receptor beta2 chain (IL-12Rβ2). It is only after the termination of TCR signaling that IL-12 dependent Th1 commitment and enhanced IFN-γ production can occur, due to IL-12 dependent up-regulation of IL-12Rβ2 (61, 62), further supporting the importance of the sequence of signals.

The late IFN-I response in EVD non-survivors resulted in concurrent IL-12 and IFN-I peaks, which, as suggested by the T cell activation model (**Figure 6**), led to lower number of effector T cells. Here, we used empirical differentiation stages, namely T_1_ and T_2_, most appropriate for describing CD8 T cells. However, both CD8 and CD4 T cells contribute to IFN-γ production. While there is evidence that IL-12 is not required for CD8 T cell proliferation (59), IL-12 has been shown to induce CD4 T cell proliferation and survival (63). Therefore, it cannot be excluded that the CD4 subset of T_2_ does undergo proliferation, which is not captured by our T cell activation model. Future studies are necessary to assess CD4 T cell proliferation along the disease course and unravel the impact of each cytokine. Thus, care should be taken when applying the T cell activation model (**Figure 5**) in other context, although we speculate that the proliferation of the CD4 subset of T_2_ is low in EVD non-survivors, due to the concurrent presence of IFN-I (64).

The analysis presented here was based on self-reported symptom onset (see Methods section), which introduced uncertainty in the real timing of infection. While a weighted bootstrap scheme (see Methods) was utilized to reconstruct an averaged version of the cytokine data for modelling, the uncertainty level associated with this approach is unclear. This limits the quantitative interpretation of the presented results. The main inference from the mathematical model — that IFN-β peaks approximately four days earlier in survivors than in non-survivors — reflects a relative timing difference; a uniform shift in the assumed incubation period displaces both groups’ DPI timelines equally and does not alter this inter-group difference. A sensitivity-relevant scenario would require survivors and non-survivors to have systematically different incubation periods; however, published data from the 2014–2016 epidemic do not support a significant difference in incubation period between the two outcome groups, and such a difference would need to exceed four days to qualitatively reverse our conclusion. Nevertheless, we acknowledge that formal sensitivity analyses regarding the incubation period assumption could further strengthen the robustness of these conclusions, representing a valuable direction for future research.

Additional limitations inherent to secondary analysis of epidemic cohort data include incomplete co-morbidity and co-infection data. Malaria co-infection status was recorded for 237 of 250 patients; 17 patients (6.8%) tested malaria-positive (**Supplementary Table 4B**). Case fatality rates were 52.9% (9/17) in malaria-positive and 40.9% (90/220) in malaria-negative patients (Fisher’s exact test: OR = 1.63, p = 0.445), suggesting a non-significant trend consistent with the known immunomodulatory effects of malaria on RANTES levels discussed above; however, with only 17 malaria-positive patients the cohort is severely underpowered to detect even a large effect or to support formal stratified analysis — we report these statistics transparently to acknowledge this limitation rather than to claim malaria has no effect. With respect to antiviral treatment, 131 of 250 patients (52.4%) participated in the JIKI favipiravir trial (**Supplementary Table 4A**). Case fatality rates were 38.9% (51/131) among trial participants and 43.7% (52/119) among non-participants — a non-significant difference (Fisher’s exact test: OR = 0.82, p = 0.520) — confirming that trial participation did not substantially shift the survival ratio in this cohort. Favipiravir acts as a nucleoside analogue inhibiting viral RNA-dependent RNA polymerase, and its primary antiviral effect is captured in the measured Ct trajectories used as empirical input to our ODE model; no separate drug-effect term is needed or appropriate. Immunomodulatory effects of favipiravir independent of its antiviral action are not documented in EVD clinical trial data (65) and are not expected to represent a significant confounder.

The concentration of RANTES tends to fluctuate significantly in malaria patients, with low levels associated with severe disease and mortality while RANTES levels increased upon recovery (66, 67). It’s important to note that some patients included in this study may have had concurrent malaria, which could potentially confound the results related to RANTES. RANTES plays a critical role in various viral infections, influencing immune response and disease outcomes, as observed in infections such as lymphocytic choriomeningitis virus (27), respiratory syncytial virus (68) and chikungunya virus (69). Our data indicated that fatal EVD patients exhibited low RANTES levels (**Supplementary Figure 2D**), which might be confounded by the presence of malaria infection. However, the decrease in RANTES levels during the recovery phase of EVD survivors contrasts sharply with the findings reported in malaria patients (66). Furthermore, RANTES levels in severe malaria were found to be lower than those in both uncomplicated malaria and healthy controls (66). In our data, RANTES levels in fatal EVD patients were higher than those of healthy controls but lower than those of EVD survivors (14). This suggests that the confounding effect of malaria on RANTES levels appears to be limited.

A key gap in current EVD patient datasets, is the absence of interferon-stimulated gene (ISG) measurements — such as MxA, OAS1, or IFIT family members — which would provide a direct, independent readout of IFN-I signaling activity in each patient and would allow a more detailed delineation of IFN regulated immune activity.

Results in this study suggested a pivotal and time-restricted role of IFN-I in organizing anti-viral response in EVD: early secretion of IFN-I leads to an orchestrated cellular response while a high level of IFN-I secreted from the same cells in the later phase leads to a distorted cellular response. The results also suggested the need for early detection of the dysfunction of the main sources of IFN-I, presumably certain dendritic cells, and corresponding pharmacological intervention, such as exogenous supplementation with IFN-I (70), for severe EVD and similar diseases. The results also suggested IL-12 as a potential biomarker for dosing stratification when considering IFN-I treatment in EVD (70). The combined machine learning and mathematical modeling approach presented in this paper might be applied to a wide range of viral infections, for example SARS-Cov-2, to get insights on the development of host immune response. The compact size of the mathematical models presented here emphasizes the beauty of fit-for-purpose small mathematical models.

## Data Availability

The data analyzed in this study were originally published by Kerber et al. (14) (PMID: 30101349). Requests for access to the raw data should be directed to the corresponding authors of that publication.
