# Supplementary material for: Delayed type I interferon response and the subsequent out-of-sequence cytokine signal inhibit T cell induction in non-surviving Ebola virus-infected patients
Source: Front Immunol. 2026 Apr 23;17:1806697. doi: 10.3389/fimmu.2026.1806697 (PMC13149275; doi:10.3389/fimmu.2026.1806697)
Supplement: Supplementary file 1 [file DataSheet1.pdf]

Supplementary code for

Delayed type 1 interferon response and the subsequent out-of-sequence cytokine signal inhibit T cell induction in non-surviving Ebola virus infected patients

Gang Zhao[1,2,*], Misa Korva[3], César Muñoz-Fontela[4], Stephan Günther[4], Romy Kerber[4], Sebastian C. Binder[1], Michael Meyer-Hermann[1,5,*]

1. Department of Systems Immunology and Braunschweig Integrated Centre of Systems Biology, Helmholtz Centre for Infection Research, Braunschweig, Germany

2. Current address: Clinical pharmacology & Quantitative pharmacology, Astrazeneca, Cambridge, UK

3. Institute of Microbiology and Immnunology, Faculty of Medicine, University of Ljubljana, Ljubljana, Slovenia

4. Bernhard Nocht Institute for Tropical Medicine, Hamburg, Germany

5. Institute for Biochemistry, Biotechnology and Bioinformatics, Technische Universität Braunschweig, Germany

Contents

Model file of ODE model (SBPD toolbox format) 2

R code for data sanity check and outlier detection 8

R code for feature importance 24

R code for generate bootstrap median of cytokine longitudinal data 30

Model file of ODE model (SBPD toolbox format)

********** MODEL NAME

M7S_g_nDrpVinput

********** MODEL NOTES

V: virus

N: IFN-beta

P: IL-12

R: RANTES

G: IFN-gamma

D: dendritic cells

********** MODEL STATES

d/dt(D) = k_D_b - r_D_d

d/dt(N) = r_N_b - dN * N

d/dt(P_simu) = r_P_b - dP * P_simu

d/dt(R) = r_R_b - dR * R

d/dt(A) = r_A_b - r_B_b - dA * A

d/dt(B) = r_B_b - dB * B

d/dt(G) = r_G_b - dG * G

D(0) = 1

N(0) = 4.0

R(0) = 58513

P_simu(0) = 0.8

B(0) = 1

G(0) = 5.5

% in steady state, rest cells are more than activated cells

A(0) = 10

B(0) = 1

********** MODEL PARAMETERS

N0 = 4.0

R0 = 58513

P0 = 0.8

G0 = 5.5

A0 = 10

B0 = 1

k_D_b = 0.0096   % steady state D = 1, half life of D is 48~72 hours in steady state

b_vn = 0.1611

H_vn = 0.01

b_vp = 0.1

H_vp= 10

b_nn = 0.03

H_nn = 49.96

b_D_d = 0.1543

H_n_D_d= 49.97

b_np = 0.1

H_np = 5

b_nr = 0.1

H_nr = 5

b_pr = 0.1

H_pr = 5

b_na = 0.01

b_aa = 0.01

H_na = 4

H_aa = 5

b_pb = 0.1

H_pb = 10

b_vg = 0.1

b_rg = 1

H_vg = 1e6

H_rg = 8

dA = 0.005

dB = 0.05 % half life 13.8 hour (41 hour (10.4049/jimmunol.171.8.3928))

dN = 0.11552 % half life 5~7 hours, I take 6 hours; 1.Zhang, K. Overview of Interferon: Characteristics, signaling and anti-cancer effect. Arch Biotechnol Biomed 1, 001?016 (2017).

dR = 0.0096   % half life 72 hour; can't find infor. I assume very slow degradation due to its huge concentration at steady state

dP = 0.0231   % 1. 5~10 hours; 2. 30 hours; I take 30 hours; 1.Carre�o, V. et al. A phase I/II study of recombinant human interleukin-12 in patients with chronic hepatitis B. J Hepatol 32, 317?324 (2000).

% 2.Robertson, M. J. et al. Immunological Effects of Interleukin 12 Administered by Bolus Intravenous Injection to Patients with Cancer. Clin Cancer Res 5, 9?16 (1999).

dG = 1.3863   % half life 0.5 hour; 1.Arnaud, P. [The interferons: pharmacology, mechanism of action, tolerance and side effects]. Rev Med Interne 23 Suppl 4, 449s?458s (2002).

sc_V = 45   % max ct is 42 (in sheet2). min ct is 14 (sheet1 and 2).

ct_ini = 50   % manually fixed. As there is no way to identify it.

%

transient_t = 0.001

flag_surv = 1

flag_dead = 0

flag_P_simu = 1

flag_P_input = 0

time_P_flip = 1e6 % simulation ends at t = 384;

********** MODEL VARIABLES

ct_input_surv = interpcsSB([0, 192, 216, 240, 264, 288, 312, 336, 360, 384], [ct_ini, 24.37, 23.84, 23.03, 23.72, 23.58, 24.89, 24.75, 26.07, 26.25], time)

ct_input_dead = interpcsSB([0, 192, 216, 240, 264, 288, 312, 336, 360, 384], [ct_ini, 19.82, 20.35, 19.57, 19.08, 18.29, 18.24, 18.71, 19.87, 20.59], time)

ct_input = flag_dead * ct_input_dead + flag_surv * ct_input_surv

```
% P of the dead

% P_input = interpcsSB([0, 192, 216, 240, 264, 288, 312, 336, 360, 384], [P0, 16.34, 17.47, 18.44, 19.33, 21.06, 25.70, 30.36, 39.21, 36.61], time)

% 25.88 is used to have smooth flip from simulation to input.

% P_input = interpcsSB([0, 192, 216, 240, 264, 288, 312, 336, 360, 384], [P0, 16.34, 17.47, 18.44, 19.33, 21.06, 25.70,   25.88, 39.21, 36.61], time)

% P = P_simu * flag_P_simu + P_input * flag_P_input

P = P_simu


V = power(10, (sc_V - ct_input) * 0.262)


n = N/N0

p = P/P0

r = R/R0

g = G/G0

a = A/A0

b = B/B0


Hill_a_vn = Hill_a(b_vn, H_vn, V, 0)

Hill_a_nn = Hill_a(b_nn, H_nn, n, 1)

r_N_b = dN * N0 * D * Hill_a_vn * Hill_a_nn


Hill_a_D_d = Hill_a(b_D_d, H_n_D_d, n, 1)

r_D_d = k_D_b   * D * Hill_a_D_d


Hill_a_vp = Hill_a(b_vp, H_vp, V, 0)

Hill_i_np = Hill_i(b_np, H_np, n, 1)

r_P_b = dP * P0    * Hill_a_vp * Hill_i_np
```

Hill_a_nr = Hill_a(b_nr, H_nr, n, 1)

Hill_i_pr = Hill_i(b_pr, H_pr, p, 1)

r_R_b = dR * R0 * Hill_a_nr * Hill_i_pr

Hill_a_na = Hill_a(b_na, H_na, n, 1)

Hill_a_aa = Hill_a(b_aa, H_aa, a, 1)

r_A_b = (dA * A0 + dB * B0) * Hill_a_na * Hill_a_aa

Hill_a_pb = Hill_a(b_pb, H_pb, p, 1)

r_B_b = dB * B0 * Hill_a_pb * a

Hill_a_vg = Hill_a(b_vg, H_vg, V, 0)

Hill_a_rg = Hill_a(b_rg, H_rg, r, 1)

r_G_b = dG * G0 * b * Hill_a_vg * Hill_a_rg

% T cell work load

wl = G/B

********** MODEL REACTIONS

********** MODEL FUNCTIONS

Hill_a_aux(b,h,s) = b + (1-b)* s*s / (s*s + h*h)

Hill_i_aux(b,h,s) = 1 - (1-b)* s*s / (s*s + h*h)

% The Hill functions pass (s0, 1), i.e. steady state level is 1.

Hill_a(b,h,s,s0) = Hill_a_aux(b,h,s) / Hill_a_aux(b,h,s0)

Hill_i(b,h,s,s0) = Hill_i_aux(b,h,s) / Hill_i_aux(b,h,s0)

sg(v1,v2,j1,j2) = 2*v1*j2/((v2-v1+j1*v2+j2*v1)+sqrt((v2-v1+j1*v2+j2*v1)*(v2-v1+j1*v2+j2*v1)-4*(v2-v1)*v1*j2))

j2solution(s,v1,v2,j1) = -s*(j1*v2+s*v1-s*v2-v1+v2)/v1/(s-1)

C2(x,y,K) = x+y+x*y / (K*K)

C3(x,y,z,K) = x+y+z+(x*y+x*z+y*z)/(K*K) + x*y*z/(K*K*K)

********** MODEL EVENTS

% e0 = ge(time, transient_t),V_simu,V1

% e0 = ge(time, transient_t+ 24 * (3+8)),flag_simu, 0, flag_input, 1

% e0 = ge(time, time_P_flip), flag_P_simu, 0, flag_P_input, 1

********** MODEL MATLAB FUNCTIONS

R code for data sanity check and outlier detection

```
---
title: "clean data from sheet1"
output:
  html_document:
    df_print: paged
---
```

This file aims at generating clean data for further analysis.

```{r setup, include=FALSE}

rm(list = ls())

library(readxl)

library(tidyverse)

library(lubridate)

library(pROC)

library(corrplot)

source("./multiplot.R")

source("./dens.R") # file from the OutlierDetection package, which does not support the new R version.

set.seed(12345)

```

```{r load data, warning=FALSE}

raw_data <- read_csv("./sheet1_rough.csv")

raw_data$X1 <- NULL

immune_feature <- c("ct", "CFH", "sFAS", "sFasL", "SAA", "CRP", "L-selectin", "MCP-2", "aHIL-29/IFN-g1", "aHM-CSF", "G-CSF", "GM-CSF", "IFNa2", "IFNy", "IL-10", "MCP-3", "IL-12p40", "IL-12P70", "sCD40L", "IL-1RA", "IL-1a", "IL-1b", "IL-2", "IL-4", "IL-5", "IL-6", "IL-8", "IP-10", "MCP-1", "MIP-1a", "MIP-1b", "TNFa", "RANTES", "sIL-1RII", "sTNFRI", "sTNFRII", "IFN-beta") %>% c("Sample_ID", "Dayspostonset", "outcome")
```

non immune features, most are hemato features

hemato_feature <- c("ct", "Antithrombin III", "ADAMTS13", "D-DIMER", "EGF", "P-SELECTIN", "PF4/CXCL4", "vWF", "sE-selectin", "Pecam-1", "Tissue Factor", "Thrombomodulin", "TPO", "sICAM-1", "sVCAM-1", "sVEGFR1", "sVEGFR2", "sVEGFR3","FERRITIN", "GAB", "TRAIL", "FRAKTALKINE", "GRO", "VEGF", "PAI-1", "tPA") %>% c("Sample_ID", "Dayspostonset", "outcome")

raw_data <- raw_data %>% set_names(~ str_replace_all(., "-", "_") %>% str_replace_all(" ","_") %>% str_replace_all("/","_"))

immune_feature <- immune_feature %>% str_replace_all("-", "_") %>% str_replace_all(" ","_") %>% str_replace_all("/","_")

hemato_feature <- hemato_feature %>% str_replace_all("-", "_") %>% str_replace_all(" ","_") %>% str_replace_all("/","_")

spec(raw_data)

` ` `

` ` `{r show some distributions, echo=FALSE}

raw_data <- filter(raw_data, !is.na(ct)) # This is very important.

p0 <- ggplot(data = raw_data) +

  geom_histogram(mapping = aes(x = ct, fill = outcome), binwidth = 1)

print(p0)

p1 <- ggplot(data = raw_data) +

```
  geom_histogram(mapping = aes(x = age, fill = outcome), binwidth = 1)

print(p1)



p2 <- ggplot(data = raw_data) +

  geom_bar(mapping = aes(x = sex,  fill = outcome))

print(p2)


raw_data$sex[which(is.na(raw_data$sex))] <- "f"       # NA replaced by median (see later) is not applicable for non-numeric ones



p3 <- ggplot(data = raw_data) +

  geom_bar(mapping = aes(x = drug_trail,  fill = outcome))

print(p3)


p4 <- ggplot(data = raw_data) +

  geom_histogram(mapping = aes(x = Dayspostonset,  fill = outcome), binwidth = 1)

print(p4)


day_max <- 10


raw_data <- filter(raw_data, Dayspostonset <= day_max)           # NA removed


p5 <- ggplot(data = filter(raw_data, outcome == "surv")) +

  geom_histogram(mapping = aes(x = `days_dateofdischarge___date_sampling`), binwidth = 1)

print(p5)
```

```
raw_data <- filter(raw_data, outcome == "surv" &
`days_dateofdischarge___date_sampling` >= 0 &
`days_dateofdischarge___date_sampling` <= 40) %>%
  bind_rows(filter(raw_data, outcome == "dead") )          # NA removed


p6 <- ggplot(data = filter(raw_data, outcome == "dead")) +
  geom_histogram(mapping = aes(x = `days_dateof_death___date_sampling`), binwidth
= 1)
print(p6)


raw_data <- filter(raw_data, outcome == "dead" &
`days_dateof_death___date_sampling` >= 0 & `days_dateof_death___date_sampling`
<= 20 ) %>%
  bind_rows(filter(raw_data, outcome == "surv"))           # NA removed


# generate new variable before_end
raw_data <- raw_data %>% mutate(
  before_end = case_when(
    outcome=="dead" ~ as.double(mdy(dateofdeath) - mdy(date_sampling)),
    outcome=="surv" ~ as.double(25 - ( mdy(dateofdischarge)  - mdy(date_sampling) ))
  )
)


p7 <- ggplot(data = filter(raw_data, outcome == "surv")) +
  geom_histogram(mapping = aes(x = before_end), binwidth = 1)
print(p7)


p8 <- ggplot(data = filter(raw_data, outcome == "dead")) +
  geom_histogram(mapping = aes(x = before_end), binwidth = 1)
```

print(p8)

```
```

```{r data processing, echo = FALSE}

raw_data[raw_data == "?"] <- NA

sumna <- function(x) sum(is.na(x))

allnacol <- as.numeric( which( apply(raw_data,2,sumna) == nrow(raw_data)) )
cat(names(raw_data)[allnacol]," have been removed because of 'all NA' \n\n")
raw_data <- select(raw_data, -all_of(allnacol))                          # remove all NA col

noinfo <- select_if(raw_data, function(.) n_distinct(.) == 1) %>%   names()
cat(noinfo, " have been removed because of 'all identical' \n\n")
raw_data <- select(raw_data, -all_of(noinfo))                            # remove all identical
column

```

Above is some general thing. Depending on the specific case, more cleaning is necessary.

````
```{r further data processing, echo = FALSE}

# remove some non useful cols
raw_data <- raw_data %>% select(-c(PlateNo, symptom_onset, date_sampling, clinical_status, Sample, test, dateofdeath ))
raw_data <- raw_data %>% select(-c(`days_dateof_death___date_sampling`, dateofdischarge,  `days_dateofdischarge___date_sampling` ))


var_many_na <- which(apply(raw_data, 2, function(x) sum(is.na(x))) >= ceiling(0.2 * nrow(raw_data)))
raw_data <- select(raw_data, -all_of(var_many_na))                          # remove cols having too many NA
cat(names(var_many_na), " have been removed because of 'high number of NA' \n\n")

ebv_data <- mutate_if(raw_data, is.numeric, list(~ replace(.,is.na(.), mean(.,na.rm= TRUE)))) # rempalce NA by mean
cat("NA have been replaced by mean of the corresponding column \n\n")


ebv_data <- ebv_data %>% mutate_if(is.character, as.factor)   # make to factor


temp_numeric <- select_if(ebv_data, is.numeric)
num_table <- map(temp_numeric, table)
max_table <- map(num_table, max)
many_identical <- which((max_table >= nrow(temp_numeric)/2 ) == TRUE)   # more than half rows are identical
````
````

```
ebv_data <- ebv_data %>% select(-all_of(names(many_identical)))        # remove
variables having large number of case with identical values

cat( colnames(temp_numeric)[many_identical], " have been excluded becuase of 'many
identical values'. \n " )

```
```

```
```{r density-based outliers in hemato_features, eval=TRUE}

data_win <- ebv_data %>% select(-sex) %>%
select(all_of(intersect(colnames(ebv_data), hemato_feature))) #

K_max <- 15

otl_h <- tribble(~K, ~outcome, ~id, ~win_s, ~win_e)

for (s in seq(0, 7)) {
 for (e in seq(4,day_max)) {
   if (e - s >= 3) {
     for (K in seq(5, K_max)) {
       numeric_dead <- data_win %>% filter(outcome == "dead") %>% select(-
c(outcome)) %>% filter(Dayspostonset >= s & Dayspostonset <=e )
       numeric_dead <- column_to_rownames(numeric_dead, var = "Sample_ID")
       try( outlier_dead <- dens(numeric_dead, k = K, rnames = TRUE) )  # OutlierDetection
is not working with the new version of R
       otl_h <- bind_rows(otl_h, tibble(K = K, outcome = 'dead', id =
rownames(numeric_dead)[outlier_dead$`Location of Outlier`], win_s = s, win_e = e))
```

```
      numeric_surv <- data_win %>% filter(outcome == "surv") %>% select(-c(outcome)) %>% filter(Dayspostonset >= s & Dayspostonset <=e )
      numeric_surv <- column_to_rownames(numeric_surv, var = "Sample_ID")
      try( {
        outlier_surv <- dens(numeric_surv, k = K, rnames = TRUE)  #

        otl_h <- bind_rows(otl_h, tibble(K = K, outcome = "surv", id = rownames(numeric_surv)[outlier_surv$`Location of Outlier`], win_s = s, win_e = e))
      })
    }
  }
 }
}

(otl_h_sum <- otl_h %>% group_by(id) %>% count() %>% arrange(desc(n))   )
```

```
```{r density-based outliers in immune_features, eval=TRUE}
data_win <- ebv_data %>% select(-sex) %>%
select(all_of(intersect(colnames(ebv_data), immune_feature)))


otl_i <- tribble(~K, ~outcome, ~id, ~win_s, ~win_e)

for (s in seq(0, 7)) {      # start of the time window
 for (e in seq(4,day_max)) {# end of the time window
  if (e - s >= 3) {
    for (K in seq(5, K_max)) { # hyperparameter
```
```

```
      numeric_dead <- data_win %>% filter(outcome == "dead") %>% select(-c(outcome)) %>% filter(Dayspostonset >= s & Dayspostonset <=e )

      numeric_dead <- column_to_rownames(numeric_dead, var = "Sample_ID")

      try( outlier_dead <- dens(numeric_dead, k = K, rnames = TRUE) )

      otl_i <- bind_rows(otl_i, tibble(K = K, outcome = 'dead', id = rownames(numeric_dead)[outlier_dead$`Location of Outlier`], win_s = s, win_e = e))


      numeric_surv <- data_win %>% filter(outcome == "surv") %>% select(-c(outcome)) %>% filter(Dayspostonset >= s & Dayspostonset <=e )

      numeric_surv <- column_to_rownames(numeric_surv, var = "Sample_ID")

      try ( {

        outlier_surv <- dens(numeric_surv, k = K, rnames = TRUE)

        otl_i <- bind_rows(otl_i, tibble(K = K, outcome = "surv", id = rownames(numeric_surv)[outlier_surv$`Location of Outlier`], win_s = s, win_e = e))

      })

    }

   }

  }

}


(otl_i_sum <- otl_i %>% group_by(id) %>% count() %>% arrange(desc(n))   )


```

```{r save whole data}
```

```
clr_data <- select(ebv_data, Sample_ID, everything())   # place sampleID to 1st col


immune_data <- clr_data %>% select(all_of(intersect(colnames(clr_data), immune_feature))) %>% filter(!(Sample_ID %in% c("GUI_004126","COY_013582","EV041","COY_013673")))

hemato_data <- clr_data %>% select(all_of(intersect(colnames(clr_data), hemato_feature))) %>% filter(!(Sample_ID %in% c("COY_013751","EV053","EV041")))

write_csv(immune_data, './sheet1_clean_immune_otl_rmv.csv')
write_csv(hemato_data, './sheet1_clean_hemato_otl_rmv.csv')

mix_data <-  clr_data %>% select(all_of(intersect(colnames(clr_data), c(immune_feature, hemato_feature)))) %>% filter(!(Sample_ID %in% c("GUI_004126","COY_013582","EV041","COY_013673"))) %>% filter(!(Sample_ID %in% c("COY_013751","EV053","EV041")))

write_csv(mix_data, './sheet1_clean_mix_otl_rmv.csv')

corrplot(cor(mix_data %>% select(-c(Sample_ID, outcome, Dayspostonset))), type="upper")
corrplot(cor(immune_data %>% select(-c(Sample_ID, outcome, Dayspostonset))), type="upper")
corrplot(cor(hemato_data %>% select(-c(Sample_ID, outcome, Dayspostonset))), type="upper")
```
```
```

```
```{r more plot2, echo=FALSE}

summ_data <- clr_data %>% group_by( WHO_ID) %>% mutate(nn =n())

p1 <- ggplot(data = summ_data) +
  geom_histogram(mapping = aes(x = nn, fill = outcome), binwidth = 1) +
  xlab("number of samples")
print(p1)

```

```{r plot }

p1 <- ggplot(data = clr_data, mapping = aes(x = Dayspostonset, y = L_selectin, color = outcome)) +
  geom_point() +
  geom_line(mapping = aes(group = WHO_ID))

p2 <- ggplot(data = clr_data, mapping = aes(x = Dayspostonset, y = `IFN_beta`, color = outcome)) +
  geom_point() +
  geom_line(mapping = aes(group = WHO_ID))

p3 <- ggplot(data = clr_data, mapping = aes(x = Dayspostonset, y = RANTES, color = outcome)) +
  geom_point() +
```
```

```
 geom_line(mapping = aes(group = WHO_ID))

p4 <- ggplot(data = clr_data, mapping = aes(x = Dayspostonset, y = `IL_12p40`, color = outcome)) +
 geom_point() +
 geom_line(mapping = aes(group = WHO_ID))



p5 <- ggplot(data = longitudinal, mapping = aes(x = Dayspostonset, y = ct, color = outcome)) +
 geom_point() +
 geom_line(mapping = aes(group = WHO_ID))

p6 <- ggplot(data = longitudinal, mapping = aes(x = Dayspostonset, y = `IFN_beta`, color = outcome)) +
 geom_point() +
 geom_line(mapping = aes(group = WHO_ID))

p7 <- ggplot(data = longitudinal, mapping = aes(x = Dayspostonset, y = RANTES, color = outcome)) +
 geom_point() +
 geom_line(mapping = aes(group = WHO_ID))

p8 <- ggplot(data = longitudinal, mapping = aes(x = Dayspostonset, y = `D_DIMER`, color = outcome)) +
 geom_point() +
 geom_line(mapping = aes(group = WHO_ID))

(dim(clr_data))
```

mylayout <- matrix(seq(1,4), ncol = 2, byrow = TRUE)

multiplot(p1, p2, p3, p4, layout = mylayout)

multiplot(p5, p6, p7, p8, layout = mylayout)

```

The figure above tells two things that I can do:

1. for the above panel, classification between "dead" and "surv".
2. for the panel below, regression, the response can be ct, or some haemorrhagic factor.

```{r more plot, echo=FALSE}

p3 <- ggplot(data = clr_data, mapping = aes(x = Dayspostonset, y = IFNy, color = outcome)) +

 geom_point() +

 geom_jitter() +

 geom_line(mapping = aes(group = WHO_ID)) #+

 #xlim(c(0, 7))

p4 <- ggplot(data = clr_data, mapping = aes(x = Dayspostonset, y = `IL_10`, color = outcome)) +

```
 geom_point() +

 geom_jitter() +

 geom_line(mapping = aes(group = WHO_ID)) #+

 #xlim(c(0, 7))

p5 <- ggplot(data = clr_data, mapping = aes(x = Dayspostonset, y = `ct`, color =
outcome)) +

 geom_point() +

 geom_jitter() +

 geom_line(mapping = aes(group = WHO_ID)) #+

 # xlim(c(0, 7)) +

 # ylim(c(0, 500))

p6 <- ggplot(data = clr_data, mapping = aes(x = Dayspostonset, y = `TNFa`, color =
outcome)) +

 geom_point() +

 geom_jitter() +

 geom_line(mapping = aes(group = WHO_ID)) #+

 # xlim(c(0, 7))

mylayout <- matrix(seq(1,4), ncol = 2, byrow = TRUE)

multiplot(p3, p4, p5, p6, layout = mylayout)

` ` `

` ` `{r logistic regression and ROC curve}

setdiff(colnames(clr_data), unique(c(immune_feature, hemato_feature)))
```

```
model <- glm(outcome ~ ., data = immune_data %>% select(-c(Sample_ID)), family =
binomial)

summary(model)


predictions <- predict(model, select(patient_1st_sample, everything()))

my_roc <- roc(patient_1st_sample$outcome, predictions)

plot.roc(my_roc, print.auc = TRUE, grid = TRUE, xlim = c(1,0))


predictions <- predict(model, select(immune_data, everything()))

my_roc <- roc(immune_data$outcome, predictions)

plot.roc(my_roc, print.auc = TRUE, grid = TRUE, xlim = c(1,0))


model1 <- glm(outcome ~ ct + Dayspostonset + sTNFRI + IFN_beta, data =
immune_data %>% select(-c(Sample_ID)), family = binomial)

summary(model1)


predictions <- predict(model1, select(immune_data, everything()))

my_roc <- roc(immune_data$outcome, predictions)

plot.roc(my_roc, print.auc = TRUE, grid = TRUE, xlim = c(1,0))


predictions <- predict(model1, select(patient_1st_sample, everything()))

my_roc <- roc(patient_1st_sample$outcome, predictions)

plot.roc(my_roc, print.auc = TRUE, grid = TRUE, xlim = c(1,0))


model2 <- glm(outcome ~ ct + `IL_12p40` + RANTES + `IFN_beta`, data =
patient_1st_sample, family = binomial)

summary(model2)
```

```
predictions2 <- predict(model2, select(patient_1st_sample,c(ct, `IL_12p40`, RANTES, `IFN_beta`)))

my_roc2 <- roc(patient_1st_sample$outcome, predictions2)

plot.roc(my_roc2, print.auc = TRUE, grid = TRUE, xlim = c(1,0))


model3 <- glm(outcome ~ ct + `IL_1RA`, data = clr_data, family = binomial)

summary(model3)

predictions3 <- predict(model3, select(clr_data,c(ct, `IL_1RA`)))

my_roc3 <- roc(clr_data$outcome, predictions3)

plot.roc(my_roc3, print.auc = TRUE, grid = TRUE, xlim = c(1,0))
```

R code for feature importance

```
# use the resampling in mlr3 to repeat the feature selection in ebola data that I have done by the inhouse function feature_selection_by_logistic_eNet.



# load data and packages -------------------------------------------------


rm(list = ls())

library(mlr3verse)

library(tidyverse)


set.seed(54321)


# data_file <- "./sheet1_clean_immune_otl_rmv.csv"
```

```
data_file <- "./sheet1_clean_hemato_otl_rmv.csv"

my_data <- read_csv(data_file)


time_tag <-  Sys.time() %>% str_replace_all(" ", "_") %>% str_replace_all(":", "_") %>%
str_replace_all("-", "_")

file_name <- if_else(str_detect(data_file, "immune"), str_c("immune_mlr3_", time_tag,
".RData"), str_c("hemato_mlr3_", time_tag, ".RData"))



# prepare for the loop ---------------------------------------------------


n_feature <- ncol(my_data) - 3 # Sample_ID, Dayspostonset, outcome


train_r <- 0.75   # ration of training my_data


cv_fold <- 4     # cross-validation folds


alpha <- 0.95


cv_r <- 1 - 1/cv_fold # ration of training in the cross validation


min_row <- n_feature / train_r / cv_r * 1.25 # 0.8 go to training set, 5 fold cross-
validation, 1.25 gives some extra level of safety


# earliest_win_end <- my_data %>% group_by(Dayspostonset) %>% count %>%
ungroup() %>% mutate(cumsum = cumsum(n)) %>% filter(cumsum > min_row) %>%
select(Dayspostonset) %>% min


earliest_win_end <- 4
```

```
latest_win_end <- 9

win_end_array <- seq(earliest_win_end, latest_win_end)

my_repeat <- 1000

# construct the pipeline -----------------------------------------------------------

  ### learner
my_lrn <- lrn("classif.cv_glmnet")

my_lrn$param_set$values <- list(nfolds = cv_fold,
                      type.measure = "auc",
                      # relax = TRUE,
                      # gamma = 1,
                      fdev = 1e-3,
                      alpha = alpha)

  ### pre-processing (not necessary, since glmnet has a (default) "standardize" action)

# my_pp <- po("scale")      # pre-processing
#
# my_pl <- my_pp %>>% my_lrn   # mlr3 pipeline

future::plan("multisession")

  # pre-allocate for result
```

```
result <- list(beta_list = lapply(1: my_repeat,
                      function(x) vector(mode = "double", length = n_feature )),
        accuracy = vector(mode = "double", length = my_repeat))

result_win <- vector(mode = "list") # loop win_end

el_time <- vector(mode = "double", length = length(win_end_array))

for (i in seq_along(win_end_array)) {

  ### my_data (task)

 my_tsk <- TaskClassif$new("my_tsk",
               backend = as.data.frame(my_data %>%
                        filter(Dayspostonset <= win_end_array[i]) %>%
                        select(-c(Sample_ID,Dayspostonset)) %>%
                        mutate_if(is.character, as.factor)),
               target = "outcome", positive = "surv")


 ### ensemble by re-sampling

 my_resmp <- rsmp("repeated_cv", repeats = my_repeat, folds = cv_fold)
 my_resmp$instantiate(my_tsk)

 time_s <- Sys.time()
```

```
  # rr <- resample(my_tsk, my_pl, my_resmp, store_models = TRUE, store_backends =
FALSE)
  rr <- resample(my_tsk, my_lrn, my_resmp, store_models = TRUE, store_backends =
FALSE)


  time_e <- Sys.time()
  el_time[i] <- time_e - time_s


  ### aggregate result


  result_win[[i]] <- future.apply::future_sapply(rr$learners, FUN = my_fun <- function(x) {
    m <- x$model
    id.lambda.min <- m$index[1]  # index has 2 ids, for lambda.min and lambda.1se,
respectively
    result$accuracy <- m$cvm[id.lambda.min]
    result$beta_list <- m$glmnet.fit$beta[,id.lambda.min]


    # id_gamma <- m$relaxed$index["min","Gamma"]
    # id_lambda <- m$relaxed$index["min","Lambda"]
    # result$accuracy <- m$relaxed$statlist[[id_gamma]]$cvm[id_lambda]
    # result$beta_list <- m$glmnet.fit$beta[,id_lambda]
    result
  })
 }




# make data for plot -----------------------------------------------------
```

```
auc_list <- vector(mode = "list")

for (i in seq_along(win_end_array)) {

  beta_list <- (result_win[[i]]["beta_list",])
  accuracy <- unlist(result_win[[i]]["accuracy",])

  outlier <- boxplot.stats(accuracy)$out

  if (!identical(numeric(0), outlier)) {
    outlier_id <- which(accuracy %in% outlier)
    accuracy <- accuracy[-outlier_id]
    beta_list <- beta_list[-outlier_id]
  }

  beta <- flatten_dbl(beta_list)
  beta <- beta[-which(near(beta,0))]

  auc_list[[i]] <- accuracy

  coef_df <- tibble(mediator = names(beta), beta = beta) %>% group_by(mediator)
  summ_df <- coef_df %>% summarise(n = n(), neg = sum(beta<0) / n, pos =
sum(beta>0)/n, mean_beta = mean(beta)) %>%
    mutate(freq = n / max(n)) %>%
    mutate(freq = freq * if_else(pos > neg, 1, -1), win_end = win_end_array[i]) %>%
arrange(desc(freq))

  if (i==1) {
    feature_imp <-summ_df
```

```
 } else{
 feature_imp <- bind_rows(feature_imp, summ_df)
 }}

save.image(file = file_name)

# prepare my_data for plot ---------------------------------------------------
```

R code for generate bootstrap median of cytokine longitudinal data

```
---
title: "generate longitudinal data for ODE"
output:
 html_document:
  df_print: paged
 html_notebook: default
 pdf_document: default
---
```

Several new variables are indicated to act against viral growth, by the feature selection appoach. I now generate the longitudinal data to see the feasibility for ODE model.

To account for the uncertaity in the Dayspostonset, I assume that data on day i is floating from i-1 to i+1, with weighted probability of 0.5, 1, 0.5 for day i-1, i, i+1 respectively. So the data on day i is composed by D_(i-1), D_i, D_i, D_(i+1). I will take the median and get the standard deviation by bootstrapping.

````
```{r setup, include = TRUE}

rm(list = ls())
library(tidyverse)
library(doParallel)

source('multiplot.R')

common_rand_seed <- 1234L
set.seed(common_rand_seed)

```


```{r load data, include=TRUE}

sheet1 <- read_csv("./sheet1_clean.csv")

# this commented one shows how before_end is generated
# raw_data <- raw_data %>% mutate(
#   before_end = case_when(
#    outcome=="dead" ~ mdy(dateofdeath) - mdy(date_sampling),
#    outcome=="surv" ~ 360 - ( mdy(dateofdischarge)  - mdy(date_sampling) )
#   )
# ) # generate new variable before_end
````
````

```
prooved_good_shift_before_end <- 29

sheet1 <- sheet1 %>% mutate( before_end = case_when(
  outcome=="dead" ~ before_end ,
  outcome=="surv" ~ before_end - 360 + prooved_good_shift_before_end))

` ` `

` ` `{r bootstrap, surv}

surv <- sheet1 %>% filter(outcome == "surv")

n_boot <- 10000

median_matrix <- matrix(data = NA, nrow = n_boot, ncol = ncol(surv) - 8)

n_cluster <- 9L
cl <- makeCluster(n_cluster)  #not to overload your computer
registerDoParallel(cl)

median_sd_surv <- foreach(day_post_onset = 1:n_cluster, .combine = rbind, .packages
= c("magrittr", "dplyr")) %dopar% {

   data_day <- bind_rows( filter(surv, Dayspostonset == day_post_onset),
                    filter(surv, Dayspostonset == day_post_onset),
                    filter(surv, Dayspostonset == day_post_onset - 1),
                    filter(surv, Dayspostonset == day_post_onset + 1))
   data_day <- data_day %>% select(-c(Sample_ID, WHO_ID, sex, age, outcome,
drug_trail, Dayspostonset, before_end))
```

```
  for (e_boot in seq(1:n_boot)) {
    id <- sample(nrow(data_day), replace = TRUE)
    data_sample <- data_day[id,]
    median_matrix[e_boot,] <- apply(data_sample, MARGIN = 2, median)
  }
  median_day <- apply(median_matrix, MARGIN = 2, median)
  sd_day <- apply(median_matrix, MARGIN = 2, sd)

  result <- matrix(c( median_day, sd_day), nrow = 2, byrow = TRUE)
  colnames(result) <- colnames(data_day)
  return(result)
}

stopCluster(cl)

` ` `

` ` `{r bootstrap, dead}

dead <- sheet1 %>% filter(outcome == "dead")

n_boot <- 10000

median_matrix <- matrix(data = NA, nrow = n_boot, ncol = ncol(dead) - 8)

n_cluster <- 9L
cl <- makeCluster(n_cluster)  #not to overload your computer
```

```
registerDoParallel(cl)

median_sd_dead <- foreach(day_post_onset = 1:n_cluster, .combine = rbind, .packages = c("magrittr", "dplyr")) %dopar% {

  data_day <- bind_rows( filter(dead, Dayspostonset == day_post_onset),
                         filter(dead, Dayspostonset == day_post_onset),
                         filter(dead, Dayspostonset == day_post_onset - 1),
                         filter(dead, Dayspostonset == day_post_onset + 1))
  data_day <- data_day %>% select(-c(Sample_ID, WHO_ID, sex, age, outcome, drug_trail, Dayspostonset, before_end))

  for (e_boot in seq(1:n_boot)) {
    id <- sample(nrow(data_day), replace = TRUE)
    data_sample <- data_day[id,]
    median_matrix[e_boot,] <- apply(data_sample, MARGIN = 2, median)
  }
  median_day <- apply(median_matrix, MARGIN = 2, median)
  sd_day <- apply(median_matrix, MARGIN = 2, sd)

  result <- matrix(c( median_day, sd_day), nrow = 2, byrow = TRUE)
  colnames(result) <- colnames(data_day)
  return(result)
}

stopCluster(cl)
```

````
```{r make into tibble }

median_sd_dead_day <- bind_cols(as_tibble(median_sd_dead), day = rep(1:9, each = 2), outcome = rep("dead", 18))

median_dead <- median_sd_dead_day[seq(1, 18, 2), ]
sd_dead <- median_sd_dead_day[seq(2, 18, 2), ]

median_sd_surv_day <- bind_cols(as_tibble(median_sd_surv), day = rep(1:9, each = 2), outcome = rep("surv", 18))

median_surv <- median_sd_surv_day[seq(1, 18, 2), ]
sd_surv <- median_sd_surv_day[seq(2, 18, 2), ]

median_data <- bind_rows(median_dead, median_surv)
sd_data <- bind_rows(sd_dead, sd_surv)

```
```{r plot cytokine}

p1 <- ggplot(data = bind_cols(select(median_data, c(day, outcome, `ct`)), rename(select(sd_data, `ct`), sd = `ct`)), mapping = aes(x = day, y = `ct`, group = outcome, color = outcome)) +
  geom_point() +
  geom_line() +
  geom_errorbar(aes( ymin = `ct` - sd, ymax = `ct` + sd))
````
````

````
p2 <- ggplot(data = bind_cols(select(median_data, c(day, outcome, `TNFa`)), rename(select(sd_data, `TNFa`), sd = `TNFa`)), mapping = aes(x = day, y = `TNFa`, group = outcome, color = outcome)) +

  geom_point() +

  geom_line() +

  geom_errorbar(aes( ymin = `TNFa` - sd, ymax = `TNFa` + sd))


p3 <- ggplot(data = bind_cols(select(median_data, c(day, outcome, `IFNy`)), rename(select(sd_data, `IFNy`), sd = `IFNy`)), mapping = aes(x = day, y = `IFNy`, group = outcome, color = outcome)) +

  geom_point() +

  geom_line() +

  geom_errorbar(aes( ymin = `IFNy` - sd, ymax = `IFNy` + sd))


p4 <- ggplot(data = bind_cols(select(median_data, c(day, outcome, `IL-10`)), rename(select(sd_data, `IL-10`), sd = `IL-10`)), mapping = aes(x = day, y = `IL-10`, group = outcome, color = outcome)) +

  geom_point() +

  geom_line() +

  geom_errorbar(aes( ymin = `IL-10` - sd, ymax = `IL-10` + sd))


mylayout <- matrix(seq(1,4), nrow = 2, byrow = TRUE)


multiplot(p1, p2, p3, p4, layout = mylayout )



```
````
````

```{r plot other cytokine}

p5 <- ggplot(data = bind_cols(select(median_data, c(day, outcome, `ct`)), rename(select(sd_data, `ct`), sd = `ct`)), mapping = aes(x = day, y = `ct`, group = outcome, color = outcome)) +
  geom_point() +
  geom_line() +
  geom_errorbar(aes( ymin = `ct` - sd, ymax = `ct` + sd))

p6 <- ggplot(data = bind_cols(select(median_data, c(day, outcome, `IL-1b`)), rename(select(sd_data, `IL-1b`), sd = `IL-1b`)), mapping = aes(x = day, y = `IL-1b`, group = outcome, color = outcome)) +
  geom_point() +
  geom_line() +
  geom_errorbar(aes( ymin = `IL-1b` - sd, ymax = `IL-1b` + sd))

p7 <- ggplot(data = bind_cols(select(median_data, c(day, outcome, `L-selectin`)), rename(select(sd_data, `L-selectin`), sd = `L-selectin`)), mapping = aes(x = day, y = `L-selectin`, group = outcome, color = outcome)) +
  geom_point() +
  geom_line() +
  geom_errorbar(aes( ymin = `L-selectin` - sd, ymax = `L-selectin` + sd))

p8 <- ggplot(data = bind_cols(select(median_data, c(day, outcome, `IL-4`)), rename(select(sd_data, `IL-4`), sd = `IL-4`)), mapping = aes(x = day, y = `IL-4`, group = outcome, color = outcome)) +
  geom_point() +
  geom_line() +
  geom_errorbar(aes( ymin = `IL-4` - sd, ymax = `IL-4` + sd))
```

mylayout <- matrix(seq(1,4), nrow = 2, byrow = TRUE)

multiplot(p5, p6, p7, p8, layout = mylayout)

` ` `
