# Supplementary material for: Delayed type I interferon response and the subsequent out-of-sequence cytokine signal inhibit T cell induction in non-surviving Ebola virus-infected patients
Source: Front Immunol. 2026 Apr 23;17:1806697. doi: 10.3389/fimmu.2026.1806697 (PMC13149275; doi:10.3389/fimmu.2026.1806697)
Supplement: Supplementary file 2 [file DataSheet2.pdf]

Supplementary file for

Delayed type 1 interferon response and the subsequent out-of-sequence cytokine signal inhibit T cell induction in non-surviving Ebola virus infected patients

Gang Zhao[1,2,*], Misa Korva[3], César Muñoz-Fontela[4], Stephan Günther[4], Romy Kerber[4], Sebastian C. Binder[1], Michael Meyer-Hermann[1,5,6*]

1. Department of Systems Immunology and Braunschweig Integrated Centre of Systems Biology, Helmholtz Centre for Infection Research, Braunschweig, Germany
2. Current address: Clinical pharmacology & Quantitative pharmacology, Astrazeneca, Cambridge, UK
3. Institute of Microbiology and Immnunology, Faculty of Medicine, University of Ljubljana, Ljubljana, Slovenia
4. Bernhard Nocht Institute for Tropical Medicine, Hamburg, Germany
5. Institute for Biochemistry, Biotechnology and Bioinformatics, Technische Universität Braunschweig, Germany
6. Lower Saxony Center for Artificial Intelligence and Causal Methods in Medicine (CAIMed), Hannover, Germany

Statistical methods explanation

1. Weighted Bootstrap Method for Longitudinal Data

Overview. To generate representative longitudinal cytokine profiles for the ODE model while accounting for uncertainty in the timing of sample collection relative to symptom onset, we employed a weighted bootstrap approach.
Rationale. The days post-onset (DPO) for each sample may contain measurement uncertainty. A sample recorded as "day 5" could biologically represent day 4, 5, or 6. To account for this temporal uncertainty, we developed a weighted resampling strategy that allows data to contribute to adjacent time points.
Weighting Scheme. For each target day i, samples from days i-1, i, and i+1 contribute with the following weights:

Time Point	Weight
Day i-1	0.5
Day i (counted twice)	1.0
Day i+1	0.5

Bootstrap Procedure.
1. For each day post-onset (1-9), construct a weighted dataset using the scheme above
2. Perform 10,000 bootstrap resamples with replacement from this weighted dataset
3. For each bootstrap iteration, calculate the median of all cytokine values
4. The final estimate is the median across all bootstrap iterations
5. The standard deviation across bootstrap iterations provides the uncertainty estimate

Output. This procedure generates "idealized" longitudinal trajectories for survivors and non-survivors, with median values and standard deviations for each cytokine at each time point. These trajectories serve as the target data for fitting the ODE model.

2. Machine Learning Feature Selection Workflow

Overview. We employed a machine learning approach to identify cytokines most predictive of survival outcome, using regularized logistic regression with elastic net penalty. The analysis was implemented using the mlr3 framework in R.

Data Preparation.

- Input data: Cleaned cytokine measurements from patient samples
- Outcome variable: Binary survival status (survivor vs. non-survivor)
- Features: Cytokine concentrations
- Excluded variables: Sample_ID, Dayspostonset, WHO_ID, age, sex (to focus on cytokine effects)

Elastic Net Regularization. We used elastic net regularization (alpha = 0.95), which combines L1 (lasso) and L2 (ridge) penalties:

- L1 component: Promotes sparsity by driving some coefficients to zero
- L2 component: Handles correlated features by distributing coefficients
- Alpha = 0.95 emphasizes lasso-like behavior while maintaining numerical stability

Cross-Validation Strategy.

- Outer resampling: 1000 repeats of 4-fold cross-validation
- Inner optimization: 4-fold CV for lambda selection (within each repeat)
- Metric: Area Under the ROC Curve (AUC)
- Lambda selection: lambda.min (value giving minimum cross-validated error)

Implementation Parameters:

mlr3 learner configuration:

• nfolds = 4 (4-fold cross-validation)
• type.measure = "auc" (optimize for AUC)
• fdev = 1e-3 (convergence threshold)
• alpha = 0.95 (elastic net mixing parameter)
• repeats = 1000 (repeated CV for robustness)

Feature Importance Calculation. For each cross-validation repeat, we recorded: (1) which features had non-zero coefficients, (2) the sign (positive/negative) of each coefficient, and (3) the magnitude of each coefficient. Features were ranked by selection frequency, coefficient consistency, and mean coefficient magnitude.

Time-Window Analysis. Feature selection was performed across multiple time windows (DPO $\leq$ 4, 5, 6, 7, 8, 9) to identify cytokines predictive of outcome at specific disease stages and consistently important features across time windows.

Output. The feature selection identified three key cytokines for the ODE model: IFN-β (type I interferon), IL-12p40 (pro-inflammatory cytokine), and RANTES (CCL5, chemokine). These were selected based on high selection frequency across CV repeats, consistent direction of association with outcome, and biological relevance to T cell activation pathway.

3. T cell activation model

The equations for the sequential model shown in Fig. 5a in the main text:

$$\frac{dT_1}{dt} = \alpha H_{nt1}^{a}(N) H_{t1t1}^{a}(T_1) - \beta T_1 H_{pt2}^{a}(P) - d_{t1} T_1 \quad \text{Eq. (S1)}$$

$$\frac{dT_2}{dt} = \beta T_1 H_{pt2}^{a}(P) - d_{t2} T_2 \quad \text{Eq. (S2)}$$

$$\frac{dG}{dt} = \gamma T_2 H_{vg}^{a}(V) H_{rg}^{a}(R) - d_g G \quad \text{Eq. (S3)}$$

The equations for the non-sequential model shown in Fig. 5b in the main text:

$$\frac{dT_1}{dt} = \alpha H_{nt1}^{a}(N) H_{t1t1}^{a}(T_1) H_{pt1}^{a}(P) - \beta T_1 - d_{t1} T_1 \quad \text{Eq. (S4)}$$

$$\frac{dT_2}{dt} = \beta T_1 - d_{t2} T_2 \quad \text{Eq. (S5)}$$

$$\frac{dG}{dt} = \gamma T_2 H_{vg}^{a}(V) H_{rg}^{a}(R) - d_g G \quad \text{Eq. (S6)}$$

The equations for the reverse-sequential model shown in Fig. 5c in the main text:

$$\frac{dT_1}{dt} = \alpha H_{pt1}^{a}(P) H_{t1t1}^{a}(T_1) - \beta T_1 H_{nt2}^{a}(N) - d_{t1} T_1 \quad \text{Eq. (S7)}$$

$$\frac{dT_2}{dt} = \beta T_1 H_{nt2}^{a}(N) - d_{t2} T_2 \quad \text{Eq. (S8)}$$

$$\frac{dG}{dt} = \gamma T_2 H_{vg}^{a}(V) H_{rg}^{a}(R) - d_g G \quad \text{Eq. (S9)}$$

Here N, P and R are the outputs from the minimal model (see Fig. 2 in the main text), V is viral load. T_1 and T_2 represent proliferating and effector T cells respectively. G denotes IFN-γ. The superscript of Hill functions denotes activation (a). The first and the second subscript of Hill functions denote the source and the target of the interaction (in lower case), respectively. The Hill function H^{a}_{t1t1} represents the proliferation of T cells. The hill coefficient of all Hill functions is fixed at 2.

Parameters in the Hill functions were fitted. The rate constants (α, β and γ) were determined by steady state conditions. The rest of the parameters is summarized in Table s2.

4. Viral Load Input.

Viral load is calculated from Ct values using: V = 10^((45 - ct_input) × 0.262), where 45 is a scaling constant and 0.262 is the log10 conversion factor. Ct values are provided as interpolated time series for survivors and non-survivors separately.

Table S1. Patient Demographics and Clinical Characteristics

Characteristic	Overall (n=147)	Survivors (n=71)	Non-survivors (n=76)
**Demographics**			
**Age (years), median (IQR)**	30.0 (19.0-40.0)	25.0 (18.0-36.5)	34.5 (22.5-45.2)

**Age range (years)**	0*-70	4-70	0-70
**Sex, n (%)**			
**Female**	80 (54.4)	43 (60.6)	37 (48.7)
**Male**	67 (45.6)	28 (39.4)	39 (51.3)
**Drug trial participation, n (%)**			
**Yes (favipiravir)**	56 (38.1)	31 (43.7)	25 (32.9)
**No**	91 (61.9)	40 (56.3)	51 (67.1)
**Initial viral load (Ct value)**			
**median (IQR)**	20.90 (17.95-24.12)	23.68 (21.29-27.81)	18.66 (16.95-20.74)
**range**	14.58-34.57	16.78-34.57	14.58-31.81
**Sample characteristics**			
**Patients, n**	147	71	76
**Total samples, n**	249	147	102
**Samples per patient, median (range)**	1 (1-8)	1 (1-8)	1 (1-5)
**First sample day post-onset, median (range)**	4 (0-28)	4 (0-14)	4 (0-28)
**Clinical outcomes**			
**Time to outcome (days), median (range)**	-	11.5 (0-35)	4.0 (0-18)
**Time to outcome (days), mean ± SD**	-	11.9 ± 5.5	4.7 ± 3.4

Data are presented as median (interquartile range) for continuous variables and n (%) for categorical variables. IQR = interquartile range (25th-75th percentile); SD = standard deviation. Ct = cycle threshold; lower values indicate higher viral load. Drug trial refers to participation in the JIKI clinical trial of favipiravir.
* Age = 0 indicates an infant (<1 year old).

Table S2: Parameter values in the minimal model and sequential T cell activation models

parameter	value	unit	meaning	ref. /note/95% credible interval from MCMC
d_g	1.3863	day^{-1}	degradation rate of IFN-γ	(Arnaud 2002)
d_n	0.1155	day^{-1}	degradation rate of IFN-β	(Zhang 2017)
d_p	0.0231	day^{-1}	degradation rate of IL-12	(Robertson et al. 1999)
d_r	0.0096	day^{-1}	degradation rate of RANTES	assumed, given its very high concentration at steady state.
d_d	0.0096	day^{-1}	~~apoptosis rate of pDC at steady state~~apoptosis rate of innate IFN-producing cells (D) at steady state	(Diao et al. 2006)
d_{t1}	0.005	day^{-1}	apoptosis rate of proliferating T cells	assumed, corresponding to 138 hours half-life
d_{t2}	0.05	day^{-1}	apoptosis rate of effector T cells	assumed, corresponding to 13.8 hours half-life
**parameter in function H^a_{vn}**				

b_{vn}	0.21			[0.19, 0.27]
h_{vn}				
surv.	0.012			[0.0024, 0.098]
non-surv.	42483			[26785, 662210]
**parameters in function H^a_{vp}**				
b_{vp}	0.017			[0.013, 0.031]
h_{vp}	60958			[11166, 98790]
**parameters in function H^a_{nn}**				
b_{nn}	0.067			[0.062, 0.090]
h_{nn}				
surv.	21.35			[15.28, 25.33]
non-surv.	18.52			[13.59, 21.29]
**parameters in function H^a_{nc}**				
b_c	0.34			[0.18, 0.36]
h_{nc}	12.09			[10.32, 30.28]
**parameters in function H^i_{np}**				
b_{np}	0.32			[0.033, 0.53]
h_{np}	6.33			[4.70, 25.82]
**parameters in function H^a_{nr}**				
b_{nr}	0.0097			[0.001, 0.028]
h_{nr}	4.27			[3.80, 6.55]
**parameters in function H^i_{pr}**				
b_{pr}	0.36			[0.24, 0.46]
h_{pr}	2.01			[1.09, 10.72]
**parameters in function H^a_{nt1}**				
b_{nt1}	0.33			[0.061, 0.49]
h_{nt1}	2.02			[2.15, 83.4]
**parameters in function H^a_{t1t1}**				
b_{t1t1}	0.0027			[0.0013, 0.0095]
h_{t1t1}	3.76			[3.68, 10.40]
**parameters in function H^a_{pt2}**				
b_{pt2}	0.048			[0.015 0.051]
h_{pt2}	41.77			[26.80, 95.56]
**parameters in function H^a_{vg}**				
b_{vg}	0.037			[0.011, 0.051]
h_{vg}	1.45e6			[1.23e6, 5.53e6]
**parameters in function H^a_{rg}**				
b_{rg}	0.39			[0.034, 0.96]
h_{rg}	961.2			[3.88, 973.81]
**initial values**				
G	5.5	pg/ml		(Kerber et al. 2018)
N	4.0	pg/ml		(Kerber et al. 2018)
P	0.8	pg/ml		(Kerber et al. 2018)
R	58513	pg/ml		(Kerber et al. 2018)
C	1			non-dimentionalized
t_1	10			assumed to be 10 folds of t_2
t_2	1			non-dimentionalized

Parameters determined by steady state conditions are: k_d, k_n, k_p, k_r (in the minimal model) and α, β, γ (in the sequential T cell activation model).

Table S3: AICc of T-cell activation models

Model	AICc*
**sequential**	39.74
**non-sequential**	318.90
**reverse-sequential**	182.30

*: a commonly used criterion for AICc in model selection is 2 units difference, i.e. models with AICc high than min(AICc)+2 can be rejected.

Table S4: Association of potential confounders with patient outcome

Table S4A. Favipiravir trial participation (JIKI trial) and patient outcome.

Favipiravir trial	Deceased (n)	Survived (n)	Total (n)	Case fatality rate (%)
Participant (yes)	51	80	131	38.9
Non-participant (no)	52	67	119	43.7
Total	103	147	250	41.2

Fisher's exact test: OR = 0.821 (95% CI not calculable by tabular method), p = 0.520.

Table S4B. Malaria co-infection status and patient outcome.

Malaria status	Deceased (n)	Survived (n)	Total (n)	Case fatality rate (%)
Positive	9	8	17	52.9
Negative	90	130	220	40.9
Not assessed	4	9	13	30.8
Total	103	147	250	41.2

Fisher's exact test (positive vs. negative only, n=237): OR = 1.625, p = 0.445. Note: the malaria-positive subgroup (n=17) is too small for stratified analysis.

Fig. S1

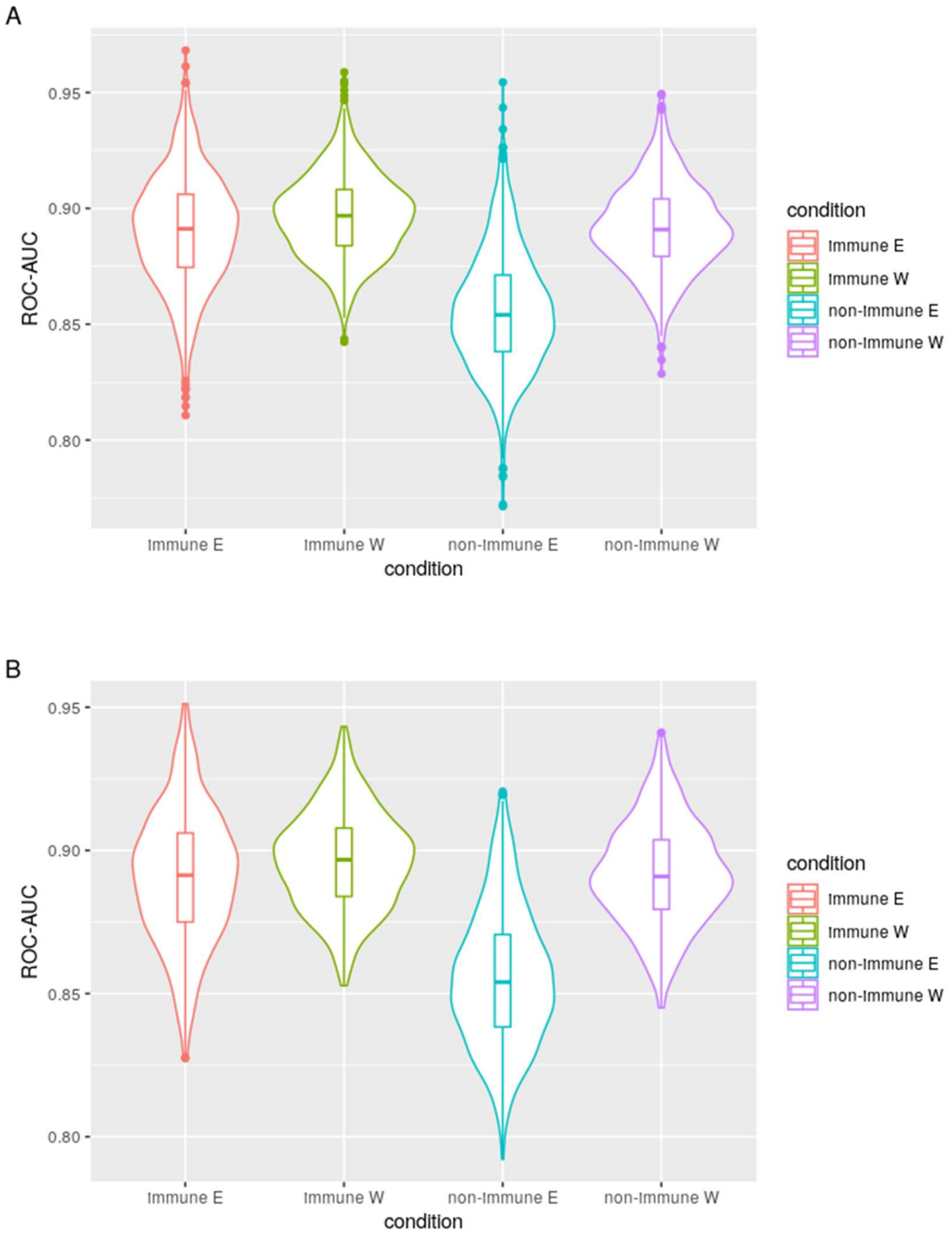

Fig. S1. Violin plot and box plot of the area under the receiver operating characteristic curve (ROC-AUC) of the ensemble learning with all results (A) or only with those in the inter-quartile range (B). The analyses were performed on immune and non-immune mediators separately, and on samples in the early (E) or whole (W) time window.

Fig. S2

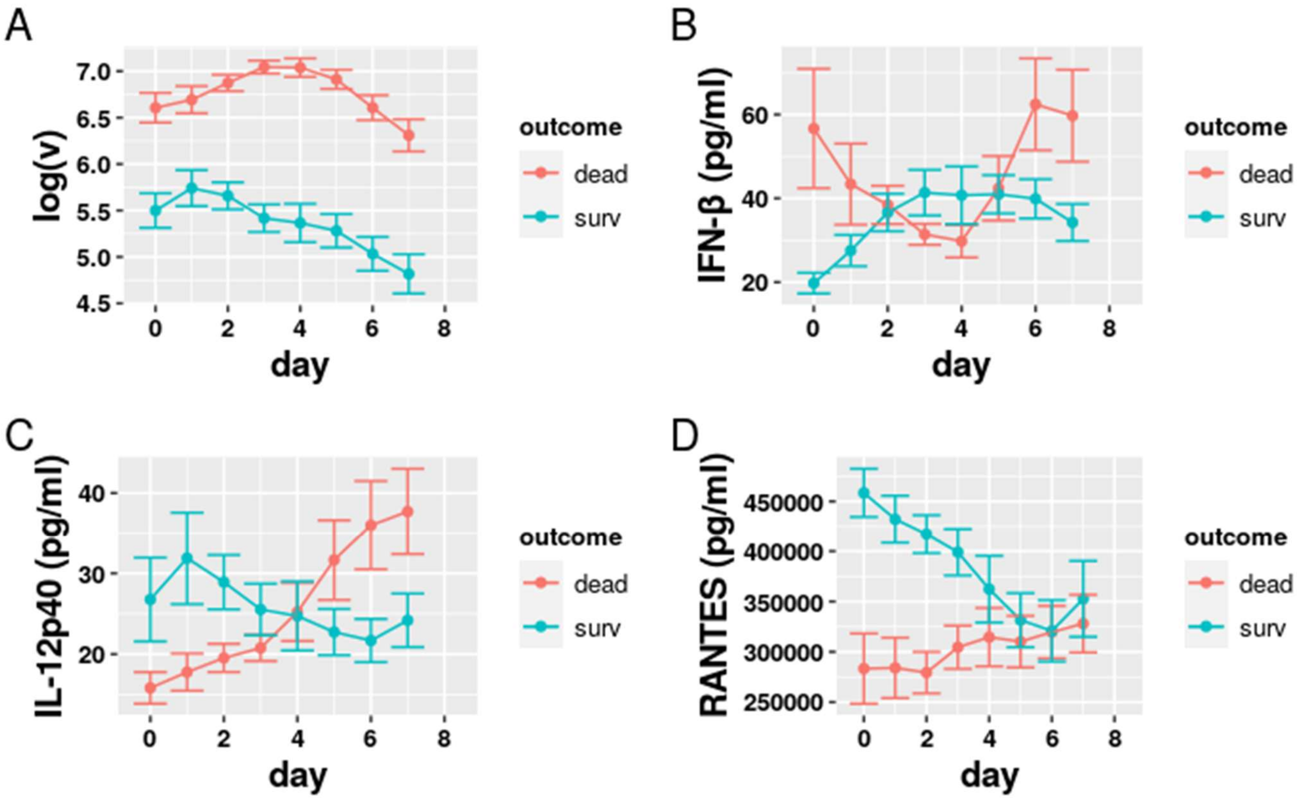

Fig. S2 The longitudinal development of the viral load, and those cytokines/chemokines that showed a switch between survivors and non-survivors. The plotted data was generated by the weighted bootstrap method. Day 0 is the time of symptom onset.

Fig. S3

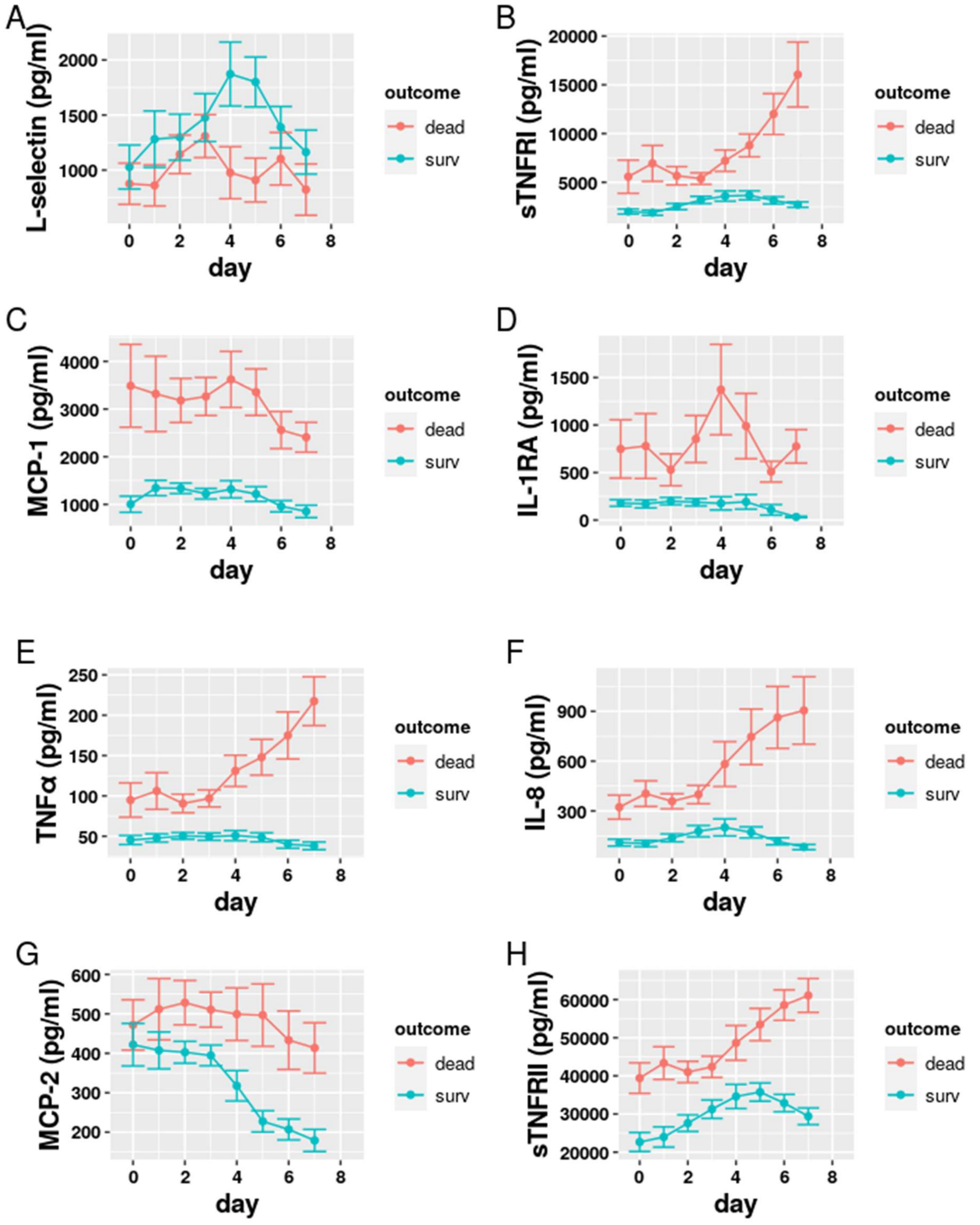

Fig. S3. The longitudinal development of those cytokines/chemokines that do not show a switch between survivors and non-survivors. The plotted data was generated by the weighted bootstrap method. Day 0 is the time of symptom onset.

Reference:

Arnaud, P. 2002. “Les différents interférons : Pharmacologie, mécanismes d’action, tolérance et effets secondaires.” *La Revue de Médecine Interne* 23 (November): 449S-458S. https://doi.org/10.1016/S0248-8663(02)00659-8.

Diao, Jun, Erin Winter, Claude Cantin, Wenhao Chen, Luoling Xu, David Kelvin, James Phillips, and Mark S. Cattral. 2006. “In Situ Replication of Immediate Dendritic Cell (DC) Precursors Contributes to Conventional DC Homeostasis in Lymphoid Tissue.” *The Journal of Immunology* 176 (12): 7196–7206. https://doi.org/10.4049/jimmunol.176.12.7196.

Kerber, Romy, Ralf Krumkamp, Misa Korva, Toni Rieger, Stephanie Wurr, Sophie Duraffour, Lisa Oestereich, et al. 2018. “Kinetics of Soluble Mediators of the Host Response in Ebola Virus Disease.” *The Journal of Infectious Diseases* 218 (Suppl 5): S496–503. https://doi.org/10.1093/infdis/jiy429.

Robertson, Michael J., Christine Cameron, Michael B. Atkins, Michael S. Gordon, Michael T. Lotze, Matthew L. Sherman, and Jerome Ritz. 1999. “Immunological Effects of Interleukin 12 Administered by Bolus Intravenous Injection to Patients with Cancer.” *Clinical Cancer Research* 5 (1): 9–16.

Zhang, Kangjian. 2017. “Overview of Interferon: Characteristics, Signaling and Anti-Cancer Effect.” *Archives of Biotechnology and Biomedicine* 1 (1): 001–016. https://doi.org/10.29328/journal.hjb.1001001.
